# Dinuclear Ir(III)-Oligomer as a Sunlight-Driven Hydroxyl Radical Generator for Effective Cancer Photodynamic Therapy

**DOI:** 10.1021/acsami.5c03823

**Published:** 2025-06-02

**Authors:** Zhao Zhang, Jinxiao Lyu, Lu Zhou, Xuanjun Zhang

**Affiliations:** Faculty of Health Sciences, 59193 University of Macau, Macau SAR 999078, China

**Keywords:** iridium(III) compound, photosensitizer, hydroxyl radical, photodynamic therapy, solid tumor

## Abstract

An interesting sunlight-driven photodynamic therapy (PDT) has been realized. In this study, we propose a strategy involving an intramolecular electron-donating ligand to develop a high-performance type-I photosensitizer. Specifically, an electron-rich core is flanked by two iridium atoms, facilitating electron transfer and promoting hydroxyl radical generation. The resulting Ir­(III) photosensitizer, Q-T, boosts the rapid generation of reactive oxygen species (ROS) under low-intensity laser exposure. Moreover, the type-I ROS ideally suits hypoxic microenvironments, thus demonstrating remarkable PDT against various cell lines, under artificial and natural sunlight. In a skin squamous carcinoma (A431) organoid model, one cycle of treatment of dosing of Q-T followed by sunlight irradiation effectively induces cellular apoptosis and completely eradicates tumor organoids. This approach offers a promising avenue for the efficient PDT of skin cancer utilizing sunlight.

## Graphical abstract

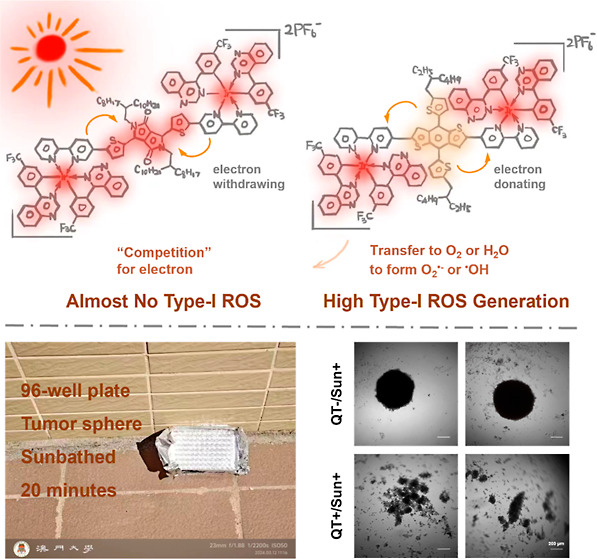

## 1 Introduction

Sunbathing is one of the most popular forms of leisure activity for most people all over the world. The practice of using sunbathing to treat diseases dates back thousands of years and has been documented in ancient Greek, Egyptian, and Indian texts.^1,2^ Modern photodynamic therapy was systematically studied only in the 1900s and was initially applied in the 1970s for bladder cancer treatment.^3,4^ Over the years, extensive research in this field has led to numerous successful applications in cancer treatment.^5–8^ However, abundant and easily accessible sunlight has often been overlooked. Exploring potential applications of sunlight-driven photodynamic therapy is indeed intriguing and interesting and revitalizes ancient magical practices with modern material sciences. Given the intensity of natural sunlight, the efficiency of photosensitizers has become a fundamental consideration. Conventionally, the heavy atom effect, which was achieved by incorporating bromide or iodine atoms into molecules to promote the intersystem crossing, has been proven effective.^9–11^ Additionally, using metal atoms to prolong triplet lifetimes enhances the generation of reactive oxygen species (ROS) upon light irradiation.^12,13^ Iridium atoms are commonly employed to construct high-performance photosensitizers, leading to remarkable therapeutic applications.^14–17^ Meanwhile, hypoxia is a common feature in solid tumors, posing a significant challenge to the efficiency of type-II photosensitizers in current photosensitizer designs.^18–21^ To address this issue, a reasonable solution involves developing type-I photosensitizers that are less dependent on oxygen to generate superoxide radicals or hydroxyl radicals.^22,23^ Among the methods used to realize this goal is the manipulation of electron transfer. One effective approach is designing electron-rich molecules, which has yielded excellent results in improving type-I photopathways.^24,25^ Additionally, the use of nanoparticles with intraparticle electron transfer has been successful.^26–28^ However, nanoparticle stability remains a concern and characterizing nanoparticles with undetermined structures presents risks. Developing molecules with well-defined structures simplifies material preparation and facilitates mechanism studies and therapeutic evaluations. Intrigued by these ideas, we have discovered that manipulation of the electron flow intramolecularly rather than intermolecularly may fulfill the strategic goal in small molecular iridium­(III) photosensitizers (see Schemes 1 and 2).

**Scheme 1:**
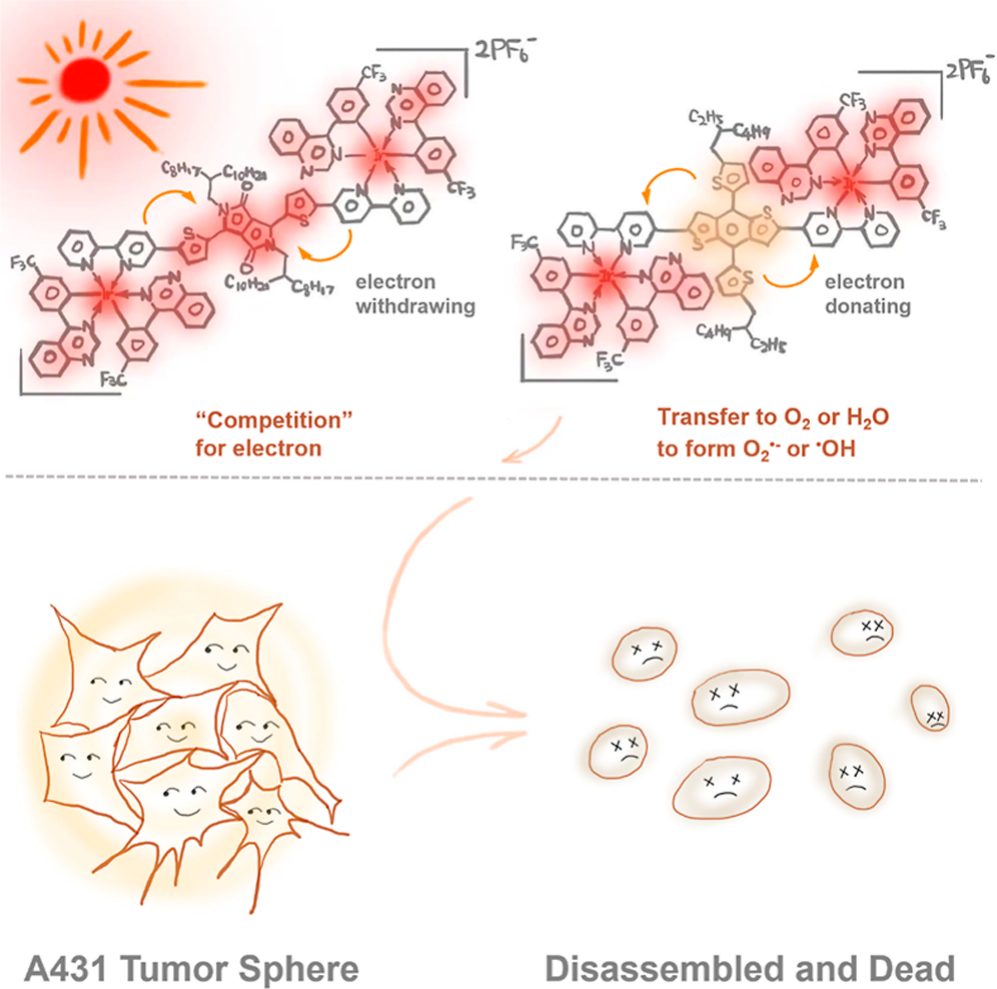
Illustration of the Hypothesis of Electron Transfer in Molecules to Generate Type-I ROS and Fulfill Efficient Cancer Cell Killing

**Scheme 2:**
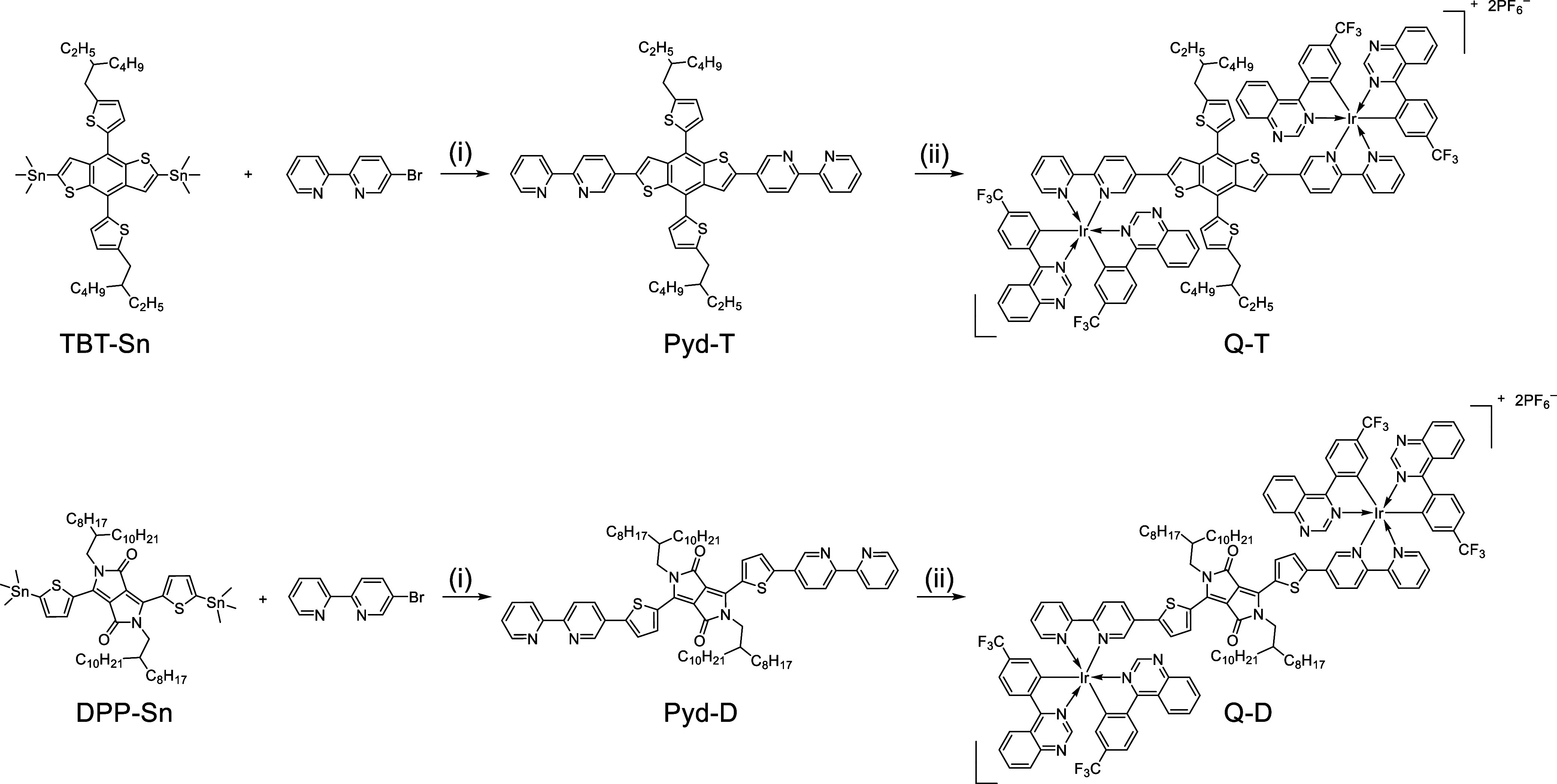
Synthesis Route of Q-T and Q-D^a^

a Reaction conditions: (i). Pd­[PPh_3_]_4_, toluene, 105 °C, 24 h; (ii) QZL2Ir-μ-Cl, silver triflate, dichloromethane/methanol (4:1), 55 °C, 12 h and then NaPF_6_, 2 h.

Our study employs an electron-rich core as an auxiliary ligand, coordinating it with two flanking iridium atoms with electron-withdrawing main ligands. Our hypothesis centers around the photosensitization process, where electrons tend to delocalize from the core to the flanking units. This electron transfer may facilitate “leakage” to other substrates such as oxygen or water, resulting in the formation of type-I ROS. We synthesized an iridium­(III) complex, denoted as Q-T and Q-D, and thoroughly investigated its photophysical and photochemical properties. Notably, both Q-T and Q-D exhibit improved absorption and photocatalysis compared to their parent molecules, lacking iridium segments (Pyd-T and Pyd-D) due to a larger absorbance cross section. Furthermore, Q-T can effectively generate type-I ROS when it is exposed to low-intensity 532 nm laser light. On the contrary, Q-D with an electron-withdrawing core can hardly generate type-I ROS. Consequently, Q-T can be activated efficiently using both artificial and natural sunlight to kill cancer cells. In an organoid model of skin squamous carcinoma (A431), one cycle of treatment with Q-T dosing and sunlight bathing leads to complete tumor sphere disintegration and nearly total eradication of cancer cells through PDT-induced apoptosis. Our findings offer straightforward and valuable guidelines for constructing high-performance type-I photosensitizers to fight against skin cancer utilizing natural sunlight bathing.

## 2 Materials and Experiments

### 2.1 Materials

All chemicals and reagents from Sigma-Aldrich were used as received from the supplier, unless otherwise stated. TBT-Sn and DPP-Sn were purchased from Derthon Optoelectronic Materials Science Technology Co., Ltd. BMPO (3,4-dihydro-2-methyl-1,1-dimethylethyl ester-2*H*-pyrrole-2-carboxylic acid-1-oxide) and TEMP (2,2,6,6-tetramethylpiperidine) were purchased from MedChemExpress. The FITC Annexin V/PI Apoptosis Detection Kit was purchased from BD Biosciences. The Calcein-AM/PI Double Stain Kit was purchased from Beyotime. A431, MCF-7, 3T3, and 4T1 cells were obtained from the Faculty of Health Science, University of Macau. Dulbecco’s modified Eagle medium, RPMI 1640 medium, fetal bovine serum, streptomycin, and penicillin were purchased from Gibco BRL.

### 2.2 Synthesis of Q-T and Q-D

#### 2.2.1 Synthesis of Pyd-T

TBT-Sn (300.00 mg, 0.331 mmol, 1.00 equiv), 5-bromo-2,2′-bipyridine (163.73 mg, 0.696 mmol, 2.10 equiv), and Pd­[PPh_3_]_4_ (57.40 mg, 49.75 μmol, 0.15 equiv) were added into a 10 mL Schlenk tube. The system was degassed by replacing the air with N_2_ three times before the addition of 3 mL of N_2_-purged blend solution of toluene. The mixture was then refluxed at 105 °C for 24 h. The mixture was concentrated under reduced pressure to afford a black crude solid. The product was purified with a silica gel column eluted with dichloromethane/methanol (100:3) to afford a black powder (268.5 mg, 91.2%).^1^H NMR (400 MHz, *d*_1_-chloroform): δ ppm: 8.99 (d, *J* = 2.0 Hz, 2H), 8.67 (d, *J* = 4.8 Hz, 2H), 8.41 (d, *J* = 8.4 Hz, 4H), 8.04 (dd, J_1_ = 2.4 Hz, J_2_ = 8.4 Hz, 2H), 7.94 (s, 2H), 7.80 (td, J_1_ = 2.0 Hz, J_2_ = 7.6 Hz, 2H), 7.38 (d, *J* = 3.6 Hz, 2H), 7.28 (m, 2H), 6.97 (d, *J* = 3.6 Hz, 2H), 2.95 (d, *J* = 6.4 Hz, 4H), 1.75 (quintet, J_1_ = 6.4 Hz, J_2_ = 6.0 Hz, 2H), 1.45 (m, 16H), 0.91 (m, 12H). ^13^C NMR (125 MHz, *d*_1_-chloroform): δ ppm: 155.70, 155.61, 146.52, 141.33, 139.37, 137.74, 137.14, 136.83, 134.31, 130.22, 128.23, 125.84, 124.02, 121.36, 121.05, 120.49, 41.69, 29.15, 26.01, 23.28, 14.42, 11.17. HRMS (MALDI-TOF, *m*/*z*) calcd for C_54_H_55_N_4_S_4_^+^ ([M]^+^), 887.3309; found, 887.3312.

#### 2.2.2 Synthesis of Compound Q-T

Pyd-T (60.00 mg, 67.62 μmol, 1.00 equiv), QZL_2_Ir-μ-Cl (109.93 mg, 71.00 μmol, 1.00 equiv), and silver triflate (32.58 mg, 135.24 μmol, 2.00 equiv) were suspended in a 5 mL solution of dichloromethane/methanol (4:1) in a 25 mL bottle. The mixture was degassed by replacing the air with N_2_ three times. The mixture was refluxed at 55 °C for 12 h, followed by the addition of NaPF_6_ (22.71 mg, 135.24 μmol, 2.00 equiv), and stirred for another 2 h. The mixture was concentrated under reduced pressure and purified with a silica gel column eluted with dichloromethane/methanol (10:1) to afford an orange powder (86.4 mg, 50.9%). ^1^H NMR (400 MHz, *d*_4_-acetone): δ ppm: 9.08­(d, *J* = 8.8 Hz, 2H), 8.99 (dd, J_1_ = 8.8 Hz, J_2_ = 15.6 Hz, 4H), 8.91 (d, *J* = 8.8 Hz, 2H), 8.82 (d, *J* = 8.4 Hz, 2H), 8.73 (t, *J* = 11.2 Hz, 4H), 8.66 (s, 2H), 8.43 (s, 2H), 8.29 (t, *J* = 8.8 Hz, 2H), 8.16 (m, 8H), 8.03 (m, 4H), 7.86 (m, 4H), 7.69 (m, 3H), 7.53 (d, *J* = 8.4 Hz, 2H), 7.46 (d, *J* = 8.8 Hz, 2H), 7.28 (d, *J* = 3.6 Hz, 2H), 7.17 (t, *J* = 8.8 Hz, 2H), 2.92 (m, 4H), 2.50 (s, 2H), 1.37 (m, 20H), 0.93 (t, *J* = 7.2 Hz, 6H), 0.85 (m, 6H). ^13^C NMR (125 MHz, *d*_4_-acetone): δ ppm: 173.65, 173.10, 154.48, 152.14, 150.30,147.51, 145.91, 136.83, 128.71, 127.21, 126.36, 125.15, 125.71, 124.62, 124.13, 122.75, 121.90, 121.44, 121.29, 119.90, 40.68, 28.27, 25.14, 22.46, 13.97, 10.61. HRMS (MALDI-TOF, *m*/*z*) calcd for C_114_H_86_F_12_Ir_2_N_12_S_4_^+^ ([M]^+^), 2364.5049; found, 2364.5056.

#### 2.2.3 Synthesis of Compound Pyd-D

DPP-Sn (300.00 mg, 0.252 mmol, 1.00 equiv), 5-bromo-2,2′-bipyridine (124.77 mg, 0.530 mmol, 2.10 equiv), and Pd­[PPh_3_]_4_ (43.81 mg, 37.91 μmol, 0.15 equiv) were added into a 10 mL Schlenk tube. The system was degassed by replacing the air with N_2_ three times before the addition of 3 mL of N_2_-purged blend solution of toluene. The mixture was then refluxed at 105 °C for 24 h. The mixture was concentrated under reduced pressure to afford a black crude solid. The product was purified with a silica gel column eluted with dichloromethane/methanol (100:3) to afford a black powder (275.5 mg, 93.2%). ^1^H NMR (400 MHz, *d*_1_-chloroform): δ ppm: 8.99 (d, *J* = 4.0 Hz, 4H), 8.68 (d, *J* = 4.0 Hz, 2H), 8.45 (m, 4H), 8.04 (dd, J_1_ = 2.4 Hz, J_2_ = 8.4 Hz, 2H), 7.82 (td, J_1_ = 1.6 Hz, J_2_ = 7.6 Hz, 2H), 7.56 (d, *J* = 4.4 Hz, 2H), 7.33 (m, 2H), 4.08 (d, *J* = 7.6 Hz, 4H), 1.98 (t, 2H), 1.25 (m, 33H), 0.83 (td, J_1_ = 2.0 Hz, J_2_ = 6.4 Hz, 6H). HRMS (MALDI-TOF, *m*/*z*) calcd for C_74_H_101_N_6_O_2_S_2_^+^ ([M]^+^), 1169.7427; found, 1169.7429.

#### 2.2.4 Synthesis of Compound Q-D

Pyd-D (40.00 mg, 34.19 μmol, 1.00 equiv), QZL2Ir-μ-Cl (54.0 mg, 34.88 μmol, 1.00 equiv), and silver triflate (16.81 mg, 69.76 μmol, 2.00 equiv) were suspended in a 5 mL solution of dichloromethane/methanol (4:1) in a 25 mL bottle. The mixture was degassed by replacing the air with N_2_ three times. The mixture was refluxed at 55 °C for 12 h, followed by the addition of NaPF_6_ (11.49 mg, 68.39 μmol, 2.00 equiv), and stirred for another 2 h. The mixture was concentrated under reduced pressure and purified with a silica gel column eluted with dichloromethane/methanol (10:1) to afford a dark-green powder (55.2 mg, 57.8%). ^1^H NMR (400 MHz, *d*_4_-acetone): δ ppm: 9.09 (d, *J* = 8.4 Hz, 4H), 9.01 (t, *J* = 8.0 Hz, 4H), 8.79 (m, 2H), 8.67 (s, 2H), 8.51 (s, 2H), 8.43 (s, 2H), 8.31 (t, *J* = 8.0 Hz, 2H), 8.17 (m, 8H), 8.04 (t, *J* = 6.8 Hz, 4H), 7.84 (m, 4H), 7.68 (t, *J* = 6.4 Hz, 2H), 7.62 (d, *J* = 8.0 Hz, 2H), 7.53 (d, *J* = 7.6 Hz, 2H), 6.77 (d, *J* = 4.8 Hz, 2H), 6.38 (s, 2H), 3.92 (s, 2H), 3.76 (s, 2H), 1.95 (s, 2H), 1.09 (m, 56H), 0.64 (m, 12H). ^13^C NMR (125 MHz, *d*_4_-acetone): δ ppm: 166.78, 160.62, 143.82, 139.77, 135.99, 132.50, 130.53, 128.96, 125.29, 126.17, 122.49, 120.07, 108.79, 45.66, 36.32, 30.38, 28.83, 22.04, 13.87. HRMS (MALDI-TOF, *m*/*z*) calcd for C_134_H_132_F_12_Ir_2_N_14_O_2_S_2_^+^ ([M]^+^) 2646.9165; found, 2646.9173.

### 2.3 Electron Paramagnetic Resonance

The EPR measurements were carried out on a Bruker Model A300 at room temperature. 200 μL of sample solution was prepared with a final concentration of 0.5 mM for photosensitizers and 100 mM for BMPO or TEMP. A white OLED light irradiated the sample at an intensity of 50 mW·cm^–2^. The signals were recorded at 0, 3, 6, and 9 min, respectively.

### 2.4 Density Functional Theory Calculation

The structures of these two molecules were optimized under the framework of density functional theory (DFT) at the (r2SCAN-3c) level, which contains the DFT-D3 dispersion correction and short-range basis correction.^29^ To investigate the photophysical properties, the excited electronic structures of these molecules were calculated with the time-dependent density functional theory (TDDFT) method. In order to calculate the scalar relativistic effect and spin–orbit coupling effect of these three molecules, which contain heavy elements of copper and iodine, the all-electron relativistic quantum chemistry calculations were performed using the DHK method and hybrid basis sets of DKH-DEF2-TZVP and SARC-DKH-TZVP; here, the SARC-DKH-TZVP basis set was used to describe the atomic orbital of iodine atoms and the DKH-DEF2-TZVP basis set was used to describe the rest of the atoms. The range-separated CAM-B3LYP functional was selected to describe the charge transfer excitation. The electron transition characterization of natural transition orbitals (NTOs) was obtained by electron excitation analysis performed using the Multiwfn program from the transition density matrix of TDDFT calculation.^30^ The spin–orbit coupling (SOC) matrix elements were calculated using spin–orbit mean-field (SOME) methods based on the excited state wave functions obtained from TDDFT calculations. All these DFT calculations above were performed using the ORCA 5.0.2 program.^31,32^ The visualization of the frontier molecular orbitals was rendered using the Visual Molecular Dynamic program (VMD).^33^

### 2.5 ROS Generation in Solution

The tetrahydrofuran solutions of DPBF (10 mM), Ce6 (1 mM), Pyd-T (1 mM), Q-T (1 mM), Pyd-D (1 mM), and Q-D (1 mM) were prepared as stock solutions. The tetrahydrofuran solution of PS was diluted in DCM to a final concentration of 2.80 μM, and the UV/vis absorption spectra were recorded. 10 μL of DPBF solution was added to the solution (final concentration was 50 μM), with its absorption spectra recorded. The mixture solution was then irradiated with a 532 nm laser with a laser power of 5 mW·cm^–2^ and its absorption spectra were recorded every 30 s. A curve of absorbance at 410 nm versus time was drawn.

### 2.6 Cell Culture Conditions

MCF-7 cells were incubated under 5.0% CO_2_ and 21.0% O_2_ at 37 °C in a humidified atmosphere in Dulbecco’s modified Eagle medium (DMEM, Gibco BRL) with 10% (v/v) fetal bovine serum (BFS, Gibco BRL) and 100 μg/mL streptomycin and penicillin (Gibco BRL). 4T1, 3T3, and A431 cells were incubated under 5.0% CO_2_ and 21.0% O_2_ at 37 °C under a humidified atmosphere in RPMI 1640 Medium (Gibco BRL) with 10% (v/v) fetal bovine serum (FBS, Gibco BRL) and 100 μg/mL streptomycin and penicillin (Gibco BRL).

For the hypoxia tests, the oxygen concentration was maintained at 1%, while all other conditions remained constant. The cells and culture medium were incubated in such a cell culture incubator. All procedures were conducted within a simple glovebox under a nitrogen atmosphere. Subsequently, the cell culture plates were sealed with plastic tape in preparation for subsequent treatment.

### 2.7 In Vitro ROS Assay

4T1 cells (50,000) were seeded in the 3.5 mL confocal microscopy dishes 16 h in advance and divided into four groups with different treatments. Q-T in DMSO was diluted with 1640 medium to a final concentration of 2 μM and used to incubate the cells in dosing groups for 2 h. All cells were incubated with H_2_DCFDA or HPF in 1640 medium without FBS for 0.5 h. The cells were further washed with PBS three times, and the laser groups were irradiated with a 532 nm laser at a power of 40 mW·cm^–2^ for 5 min. Then the PBS was replaced with fresh PBS, and confocal fluorescence images were taken soon after the laser irradiation using a Nikon A1R (λ_ex_ = 488 nm, λ_em_ = 515–535 nm).

For the ROS assay by using flow cytometry, similar treatments were performed, as described above. Then all cells were washed with fresh PBS, digested with trypsin, and collected for further detection and analysis using a flow cytometer. 10,000 cells were analyzed for flow cytometry.

### 2.8 Cell Viability Assay

A431 cells were seeded in 96-well plates at a density of 5,000 cells per well (0.1 mL) and allowed to adhere for 12 h prior to further treatment. Subsequently, the cells were incubated with varying concentrations of Q-T for 2 h. Following incubation, the cells were washed three times with PBS before undergoing irradiation with either a Xe lamp or natural sunlight for 20 min. In contrast, the cells in the dark group were washed but not exposed to irradiation. After irradiation, all cells were replenished with fresh RPMI 1640 medium and maintained in culture for an additional 24 h. The medium was then replaced with an RPMI 1640 complete medium supplemented with 10% CCK-8 solution (Beyotime Biotechnology). Following a 3 h incubation period, the absorbance at 450 nm for each well was measured using a microplate reader. The resulting absorbance values were utilized to determine cell survival rates through blank correction and normalization, and the half-maximal inhibitory concentration (IC_50_) was calculated via nonlinear regression analysis using GraphPad software.

### 2.9 Tumor Spheroid Culture

A431 cells in the logarithmic growth phase were harvested and digested with trypsin. The cells were then resuspended in a complete DMEM and seeded into ultralow attachment round-bottom 96-well plates at a density of 20,000 cells per well in 150 μL of medium. The plates were centrifuged at 200*g* for 3 min to facilitate cell aggregation. The cells were cultured under static conditions for 2 days, with 100 μL of medium replaced every other day.

On day 4, the spheroids reached a diameter of approximately 0.5 mm, at which point the Q-T was added to the culture medium at a final concentration of 5 μM. The cultures were incubated for 12 h, after which the light-irradiated group was exposed to sunlight for 20 min. After 12 h of incubation, the spheroids were stained using appropriate assay kits and analyzed under a microscope for subsequent observations.

## 3 Results and Discussion

### 3.1 Synthesis

Stille reactions are employed to efficiently synthesize Pyd-T and Pyd-D in high yields. It is worth noting that while the typical usage of the palladium catalyst in Stille reactions ranges from 0.01 to 0.03 equiv, achieving a high reaction rate and yield in this case requires 0.15 equiv of Pd­[PPh_3_]_4_. A plausible explanation for this necessity is that bipyridine may coordinate with the catalyst, rendering it inactive. The synthesis of QZL2Ir-μ-Cl follows the methodology outlined in our previous report.^14^ Its coordination reaction with bipyridine proceeds smoothly under mild conditions; however, to ensure that the reaction reaches completion, overnight processing is required, with progress monitored via thin-layer chromatography (TLC).

### 3.2 Photophysical and Photochemical Spectra

As shown in Figure 1a, due to the extension of the π conjugation, Q-T shows a higher absorption coefficient and red-shifted absorption regions, which may favor photocatalysis. Notably, Pyd-T is highly fluorescent while Q-T’s fluorescence is dramatically suppressed, indicating that some nonradiative processes take place, which may also favor the photosensation process. Similar phenomena are observed for Pyd-D and Q-D, which are summarized in Figure S1a. The ROS generation efficiency is evaluated with DPBF (1,3-diphenylisobenzofuran), whose typical absorption at 411 nm decreases in the presence of ROS. In Figure 1b,c, Q-T boosts high ROS generation much higher than that of Pyd-T, which is mainly attributed to the iridium atoms, even higher than Ce 6 as a reference photosensitizer, as shown in Figure S1b. Incorporation of the iridium atom into Pyd-D also improves ROS generation efficiency, as presumed in Figure S1c,d. Another ^•^OH probe, HPF (hydroxyphenyl fluorescein), is used to scavenge the specific ROS and turned on with fluorescence intensity increased times higher,^34^ indicating the ^•^OH generation in Q-T solution upon laser irradiation. On the contrary, as shown in Figure S1f, the ROS generated in the testing system fails to turn on HPF since almost no fluorescence increase is observed around 525 nm, indicating that no type-I species are detected. We even observed the fluorescence decrease in Q-D, which indicates problematic photostability. Further EPR (electron paramagnetic resonance) spectral measurements show Q-T generates both ^1^O_2_ and ^•^OH in the testing condition, which is consistent with former solution test results. In short, constructing Q-T improves the absorption behavior and greatly enhances the ROS generation and, more importantly, type-I ROS generation. As a result, Q-T was believed to be a brilliant type-I photosensitizer and was then studied for anticancer applications.

**Figure 1:**
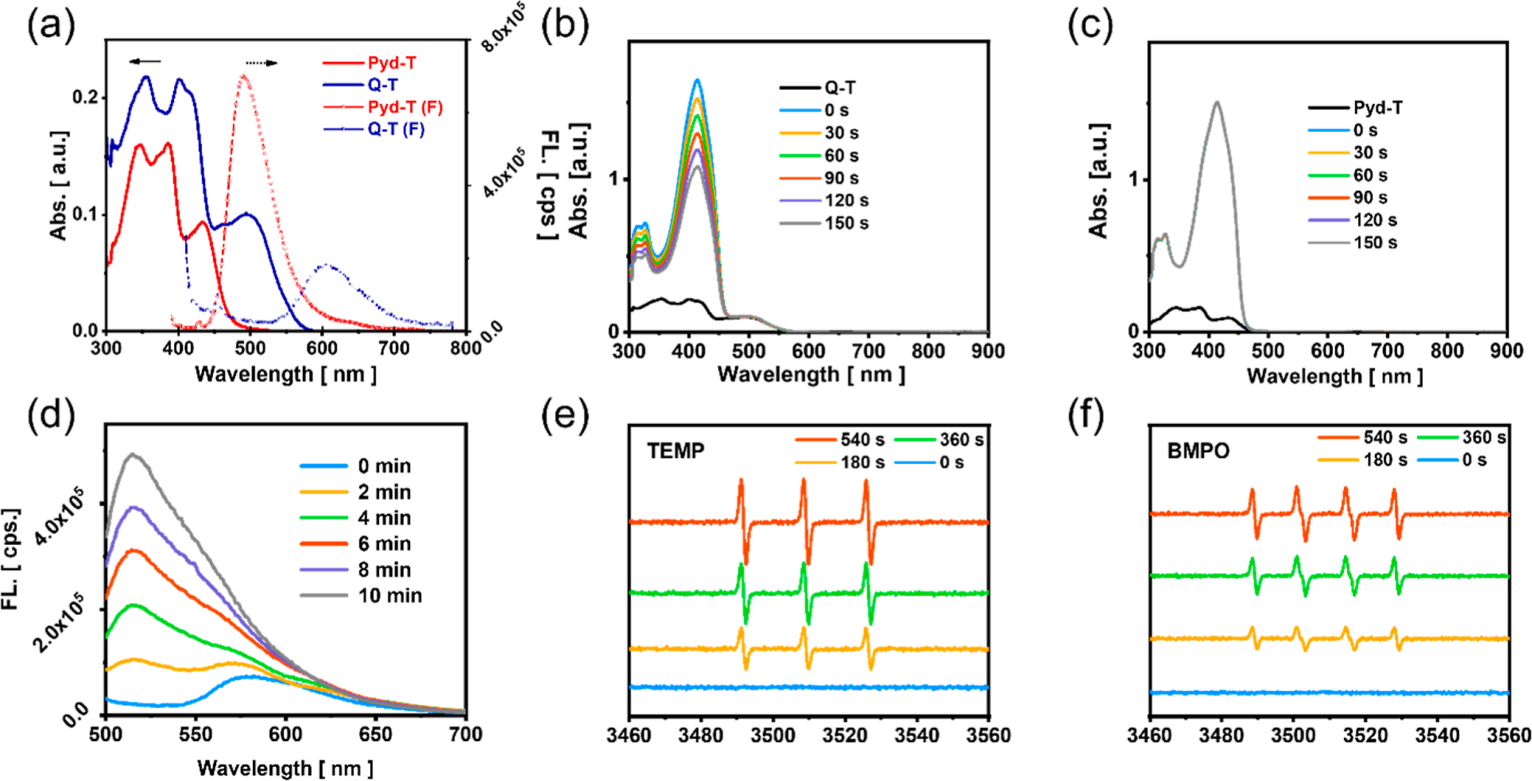
Spectral study in solution. (a) The absorption and fluorescence spectra of Q-T in dichloromethane solutions. Experimental conditions: [Q-T] = [Pyd-T] = 2.8 μM for UV/vis (solid line) and 0.56 μM for emission spectra (dash line). The absorption spectra of DPBF in dichloromethane solution. Experimental conditions: [DPBF] = 50 μM, [Q-T] = [Pyd-T] = 2.8 μM with a 532 nm laser (5 mW·cm^–2^) and recorded every 30 s. (b) Q-T and (c) Pyd-T. (d) Fluorescence spectra of HPF in the presence of Q-T irradiated with a 532 nm laser (5 mW·cm^–2^). EPR spectroscopy for Q-T with different ROS scavengers, (e) TEMP and (f) BMPO.

### 3.3 Theoretical Calculation

To enhance comprehension regarding the influence of electron-donating and electron-withdrawing ligands, density functional theory (DFT) calculations were performed based on the Q-T and Q-D molecules. The branched alkyl chains of these molecules were simplified by using methyl groups. The frontier molecular orbitals of the photosensitizers are illustrated in Figure 2. As depicted in Figure 2a,b, the electron predominantly resides in the highest occupied molecular orbital (HOMO) of the fused thiophene rings in Q-T, attributable to the electron-rich nature of these rings. Additionally, about 0.847% of the electrons are distributed on the iridium atom, while this number for Q-D decreases to 0.489% since the diketopyrrolopyrrole unit exhibits pronounced electron-withdrawing characteristics. In the excited state of both molecules, as shown in Figure 2c,d, the electrons are distributed across the entire backbone of the auxiliary ligands. Notably, the iridium atoms in Q-T demonstrate a much increased electron density (7.885% of total electrons) relative to the iridium atoms in Q-D (3.858%). These results support the hypothesis that upon excitation, the Q-T molecule is more likely to generate “hot” electrons on the iridium catalytic center and then capable of leaking into the surrounding substrate, thereby inducing the formation of type-I ROS. Importantly, the results of the DFT calculations align well with previously established findings concerning ROS generation investigation.

**Figure 2:**
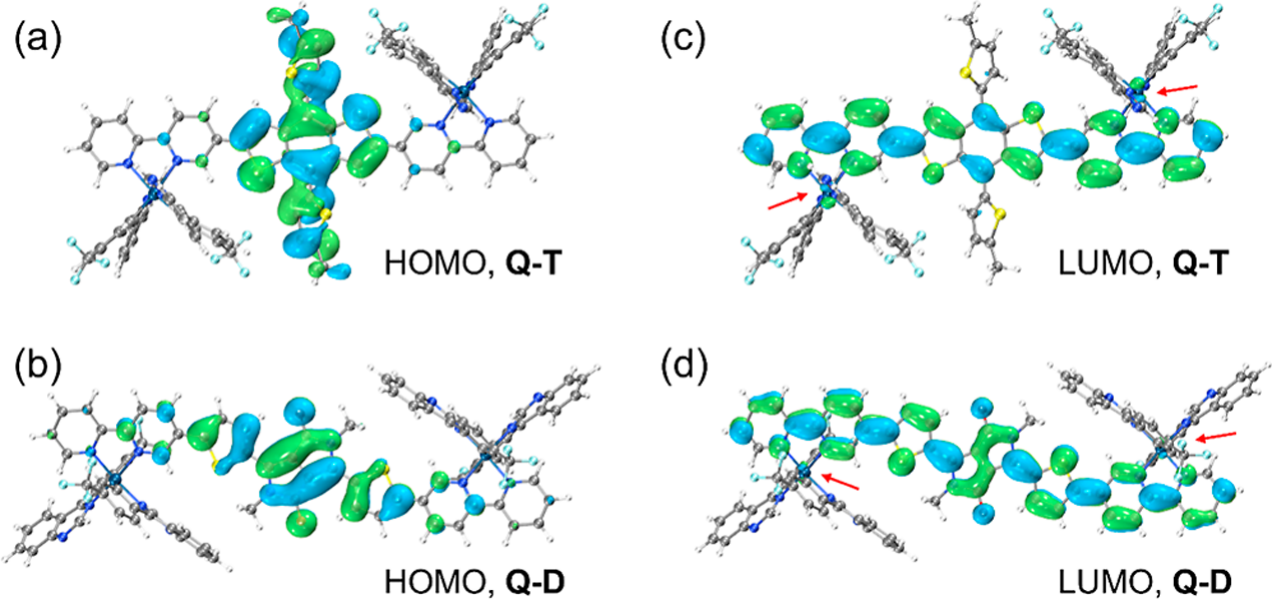
DFT-optimized geometries and frontier molecular orbitals of (a) HOMO for Q-T, (b) HOMO for Q-D, (c) LUMO for Q-T, and (d) LUMO for Q-D. The branched alkyl chains on both molecules were replaced by methyl groups.

### 3.4 Intracellular ROS Assay

The intracellular generation of ROS is confirmed with a fluorescence probe, H_2_DCFDA (2′,7′-dichlorodihydrofluorescein diacetate), in confocal imaging and flow cytometry. Only the Q-T+/L+ group detected the bright-green fluorescence of the oxidized probe (DCF), indicating a great amount of ROS is generated in cells, as shown in Figure 3a–d, while the fluorescence remained unchanged in the other three groups, in Figure S2. More detailed statistics show the trend in four groups by monitoring the green fluorescence channel, as the flow cytometry results are presented in Figure 3e. Even in hypoxic conditions, Q-T’s ROS generation behavior is not impeded, strongly indicating the type-I photochemical pathways, as shown in Figure S3. To further confirm the type-I ROS generation, one ^•^OH specific probe, HPF, was used as well. In Figure 3f–i, the probe was turned on after Q-T dosing and laser irradiation, while fluorescence remained unchanged in other groups, as shown in Figure S4. Flow cytometry results showed a similar observation in Figure 3j with probes in other groups remaining almost unaffected. A great amount of ROS, more specifically ^•^OH, can be efficiently generated by irradiating Q-T in cells.

**Figure 3:**
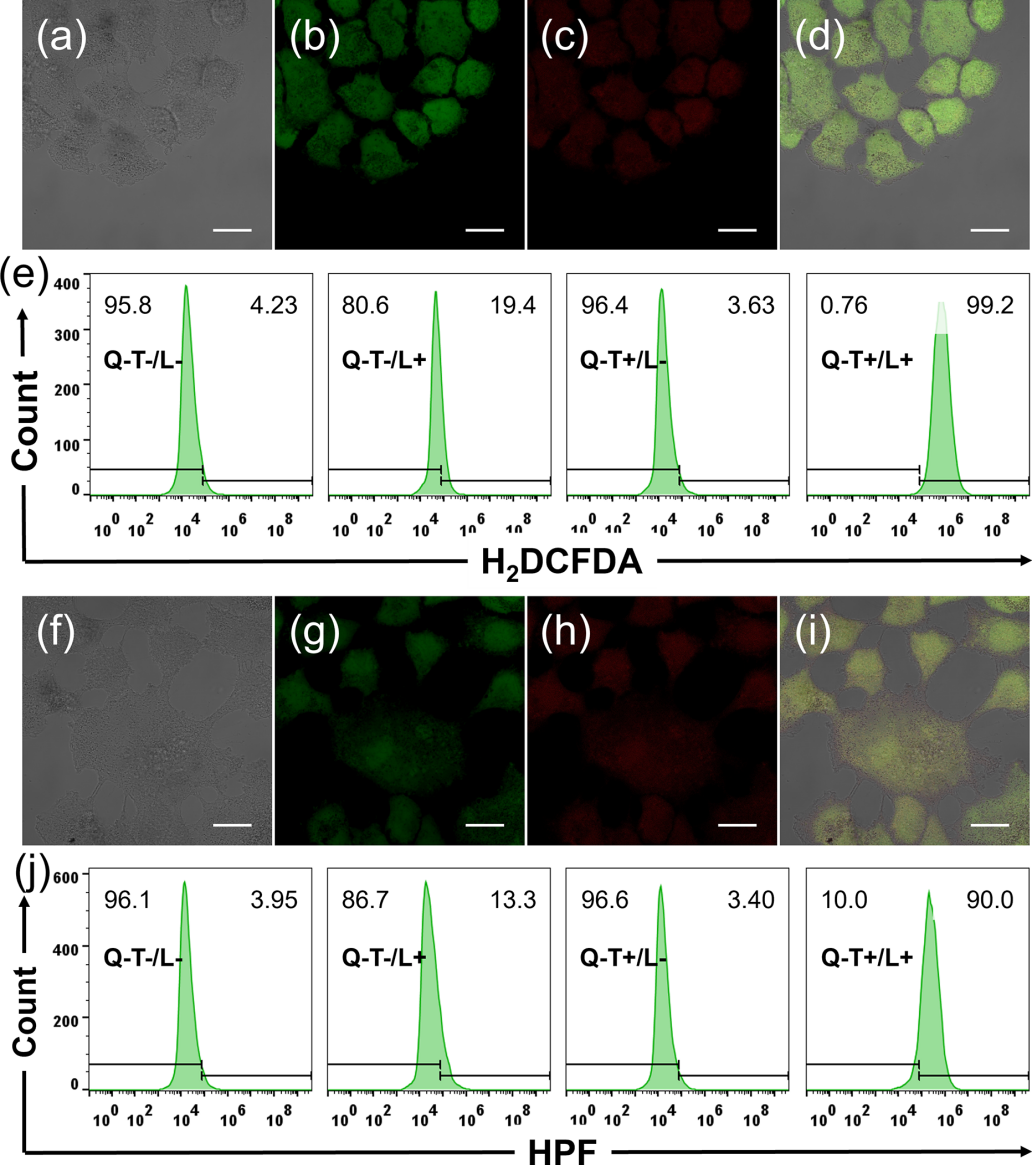
ROS assay in vitro with H_2_DCFDA (2′,7′-dichlorodihydrofluorescein diacetate) and HPF. Treatment for cells in each group, cells are cultured with Q-T (2 μM) for 2 h, and H_2_DCFDA or HPF (5 μM) for 30 min, followed by 532 nm laser irradiation (40 mW·cm^–2^) for 5 min. (a–d) Bright channel, FICT (fluorescein isothiocyanate) channel, PI (propidium iodide) channel, and merged channel for H_2_DCFDA probing. (e) Flow cytometry for total ROS evaluation for all groups. (f–i) Bright channel, FICT channel, PI channel, and merged channel for HPF probing. (j) Flow cytometry for ^•^OH evaluation for all groups. The scale bar is 20 μm.

### 3.5 Cancer Cell-Killing Evaluation

We further tested the therapeutic effects of Q-T in vitro. Q-T exhibits high cell-killing ability in several cancer cell lines, including MCF-7, 3T3, and 4T1 cells, in the presence of both the Xe lamp and natural sunlight, as shown in Figure S5. The light-driven cell killing can still be observed, even though these cells show low tolerance to natural sunbathing conditions. Skin cancer cell line A431 is proven to have a large capacity for potentially lethal damage,^35,36^ and they can effectively repair DNA damage caused by radiation exposure,^37^ and then this cell line maintains higher natural sunlight tolerance and provides strong support for efficient Q-T therapy with sunlight bathing. In Figure 4a, after Q-T treatment, sunbathed cells show decreased cell viability, and the estimated IC_50_ for Q-T is 0.429 μM (95% CI: 0.3680 to 0.5005, *N* = 4), with a PDT index over 20 (IC50_dark_ vs IC50_light_), which is sufficient for photodynamic therapy application. It is worth noting that Q-T’s PDT efficacy merely depends on the oxygen concentration (in normoxic and hypoxic conditions), as shown in Figure S6a,b. Additionally, Q-T shows much better therapeutic results than Ce6, a conventionally used, commercially available photosensitizer, which is dependent on the oxygen concentration, as shown in Figure S6c,d. In Figure 4b_1_–b_4_, further cell staining with the PI/Annexin V kit shows that PDT-induced cell apoptosis is the leading cause of the cell killing, and more detailed statistical analysis of the fluorescence reveals the high efficacy of Q-T to realize brilliant tumor cell eradication, as presented in Figure 4c.

**Figure 4:**
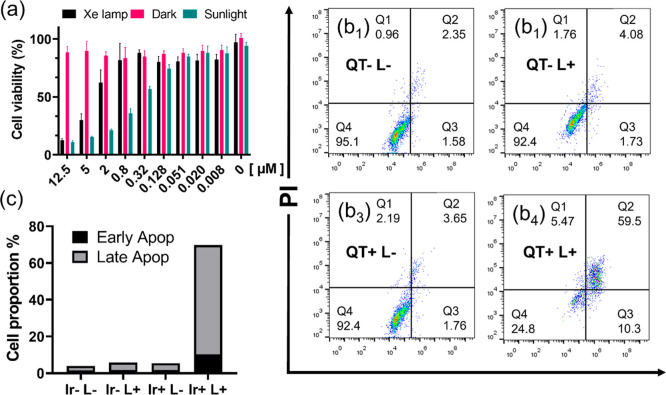
Tumor cell-killing assay. (a) A431 cell lines were treated with various concentrations of Q-T and different light conditions. Error limits on data in Table S1. (b_1_–b_4_) Cell apoptosis investigation with different treatments. (c) Statistical analysis of cell apoptosis.

The skin squamous carcinoma organoid model is used to more accurately simulate the solid tumor microenvironment for Q-T-based PDT. After Q-T dosing and 20 min of sunlight bathing for only one cycle, it is thrilling to observe that the tumor sphere is shattered into small pieces with almost no living cells left in Figure 5d_1_–d_3_. The cell apoptosis pathway is confirmed in Figure 5h_1_–h_3_. These results are consistent with the former investigation and exhibit the great potential of Q-T in PDT using natural sunlight bathing to cure skin-related cancer.

**Figure 5:**
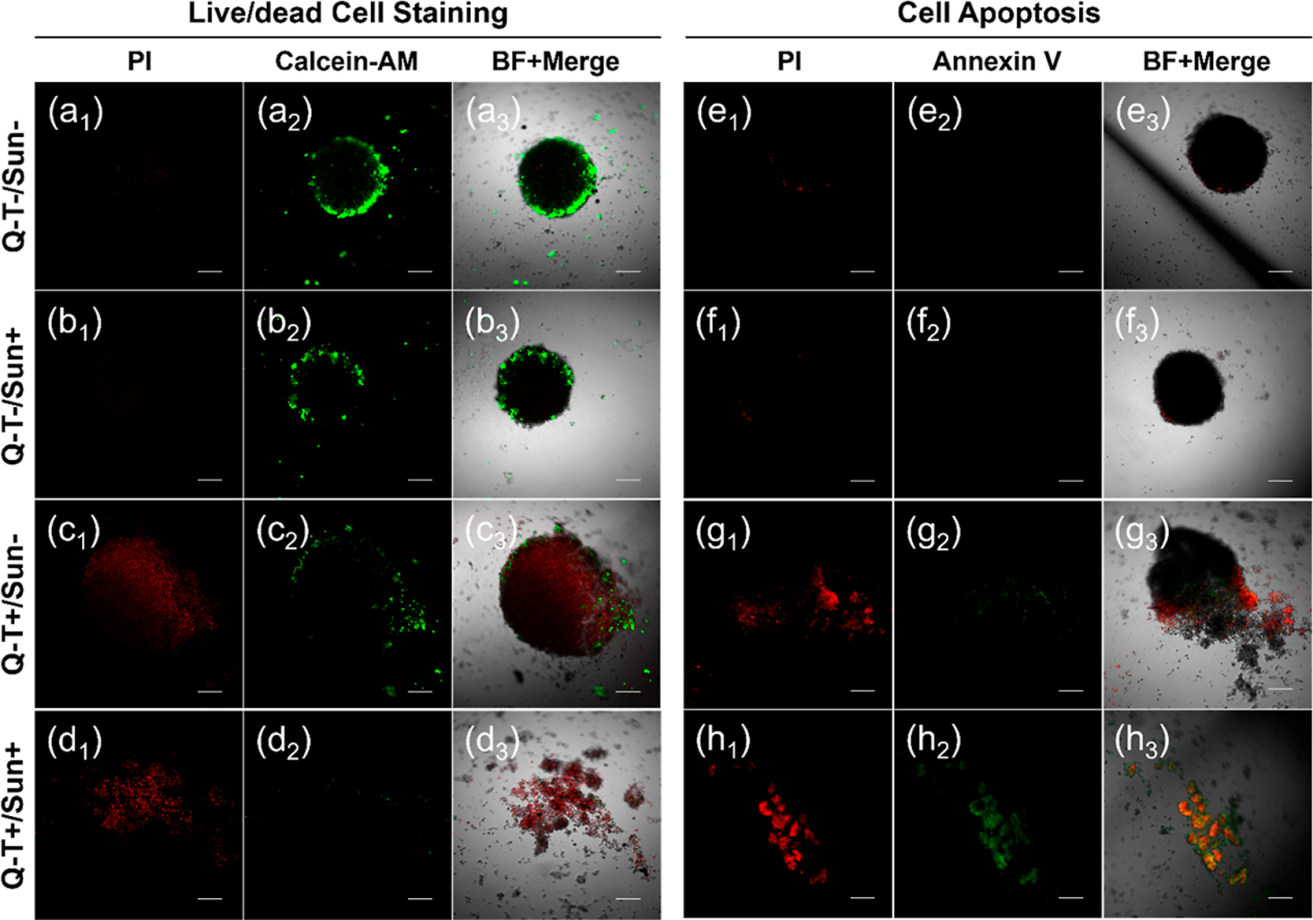
Tumor cell-killing evaluation in the solid tumor sphere. Live/dead staining with the PI/Calcein-AM kit and cell apoptosis investigation with the PI/Annexin V kit. Live/dead staining imaging: (a_1_–a_3_) control group in PI/FITC/merged channels. (b_1_–b_3_) Q-T–/sunlight+ group. (c_1_–c_3_) Q-T+/sunlight– group. (*d*_1_–*d*_3_) Q-T+/sunlight+ group. Cell apoptosis imaging: (e_1_–e_3_) control group in PI/FITC/merged channels. (f_1_–f_3_) Q-T-/sunlight+ group. (g_1_–g_3_) Q-T+/sunlight– group. (h_1_–h_3_) Q-T+/sunlight+ group. Experimental conditions: dosing groups are treated with [Q-T] = 2 μM with preincubation for 2 h and light groups are irradiated with natural sunlight for 20 min. Scale bar: 200 μm. All experiments are performed according to the standard operating protocols of each kit.

## 4 Conclusions

In summary, our study introduces a valuable strategy involving the intramolecular electron-donating ligand to promote electron transfer to the iridium moiety for designing high-performance type-I photosensitizers. These photosensitizers can be readily activated by using low-intensity artificial sunlight or natural sunlight. Compound Q-T exhibits remarkable properties. Q-T generates substantial amounts of ROS, surpassing the performance of its counterpart Pyd-T, which remains poorly activated. More importantly, the ROS produced by Q-T is confirmed to be ^•^OH, a highly toxic type-I ROS variant. This characteristic aligns well with the hypoxic tumor microenvironment, making Q-T a promising candidate for cancer therapy. Consequently, with a low IC_50_, Q-T efficiently eradicates tumor cells when it is exposed to both artificial and natural sunlight. Our research provides a practical example of harnessing abundant and easily accessible natural sunlight to combat skin cancer and address related skin diseases.

## Supplementary Material



## Notes

Photophysical properties of Q-D and ROS detection in solution; in vitro ROS assay with H_2_DCFDA and HPF; cell viability for different cell lines; and ^1^HNMR, ^13^CNMR, and MALDI-TOF spectra of the materials used (PDF)
