# Supplementary material for: Dinuclear Ir(III)-Oligomer as a Sunlight-Driven Hydroxyl Radical Generator for Effective Cancer Photodynamic Therapy
Source: ACS Appl Mater Interfaces. 2025 Jun 2;17(23):33465–73. doi: 10.1021/acsami.5c03823 (PMC12163885; doi:10.1021/acsami.5c03823)
Supplement: Supplementary file 1 [file am5c03823_si_001.pdf]

Supporting Information

Dinuclear Ir(III)-oligomer as Sunlight-driven Hydroxyl Radical Generator for effective Cancer Photodynamic Therapy

*Zhao Zhang, Jinxiao Lyu, Lu Zhou, Xuanjun Zhang**

Zhao Zhang - Faculty of Health Sciences, University of Macau, Macau SAR 999078, China

Jinxiao Lyu - Faculty of Health Sciences, University of Macau, Macau SAR 999078, China

Lu Zhou - Faculty of Health Sciences, University of Macau, Macau SAR 999078, China

Xuanjun Zhang - Faculty of Health Sciences, University of Macau, Macau SAR 999078, China; Email: xuanjunzhang@um.edu.mo

Materials and Measurements

All chemicals and reagents from Sigma–Aldrich were used as received from the supplier unless otherwise stated. ^{1}H and ^{13}C NMR spectra were measured on a Bruker AV-400 MHz NMR spectrometer with chemical shift reported in parts per million (ppm, δ). Mass spectra were recorded using the Bruker Microflex MALDI-TOF system. Uv-Vis absorption spectra were measured on a Shimadzu UV-1800 spectrometer. Photoluminescence spectra were conducted on the Horiba Fluorolog-3 spectrofluorometer. The cell culture serum and medium were purchased from Gibco. The FITC Annexin V/PI Apoptosis Detection Kit was purchased from BD Biosciences. The Calcein-AM/PI Double Stain Kit was purchased from Beyotime. A431, MCF-7, 3T3, and 4T1 cells were obtained from the Faculty of Health Science, University of Macau. All the cells were incubated in Thermo Fisher Forma Series 3 Water Jacketed CO_2 incubator. Confocal laser scanning microscope (CLSM) was performed on the Nikon A1R Confocal System.

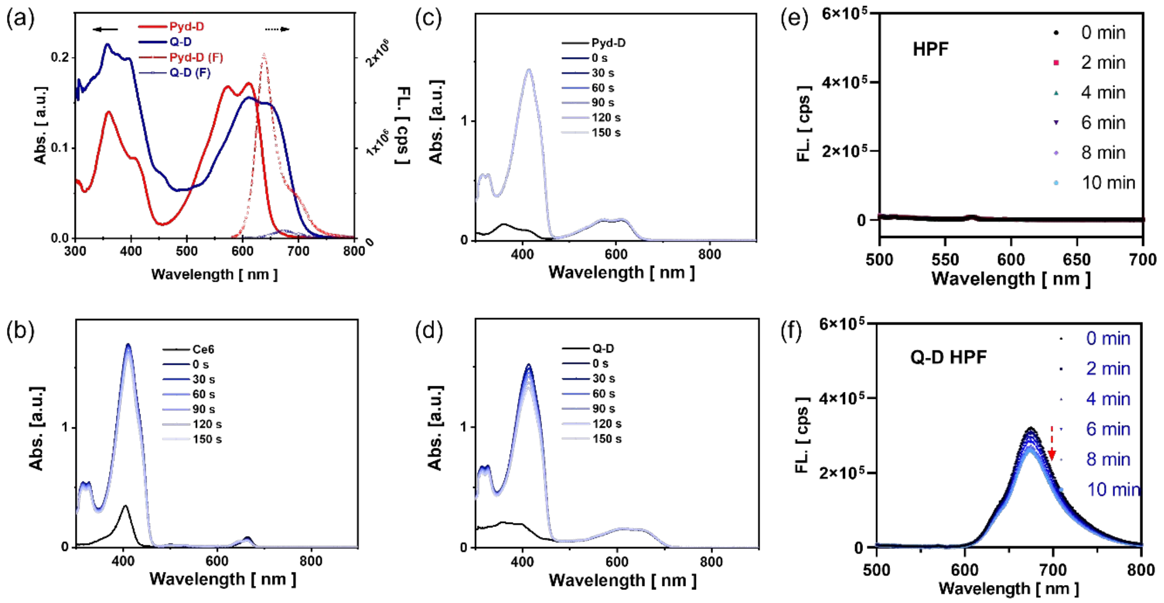

Figure S1. Spectral study in solution. (a). The absorption and fluorescence spectra of Q-D in dichloromethane solutions. Experimental conditions: [Ce 6] = [Q-D] = [Pyd-D] = 2.8 μM for Uv/Vis (solid line) and 0.56 μM for emission spectra (dash line). The absorption spectra of DPBF in dichloromethane solution. Experimental conditions: [DPBF] = 50 μM, [Q-D] = [Pyd-D] = 2.8 μM with a laser (5 mW·cm^{-2}) and recorded every 30 s. (b). Ce 6, 532 nm laser; (c). Pyd-D, 680 nm laser. (d). Q-D, 680 nm laser. (e). Fluorescence spectra of HPF. (f). Q-D and HPF, 680 nm laser.

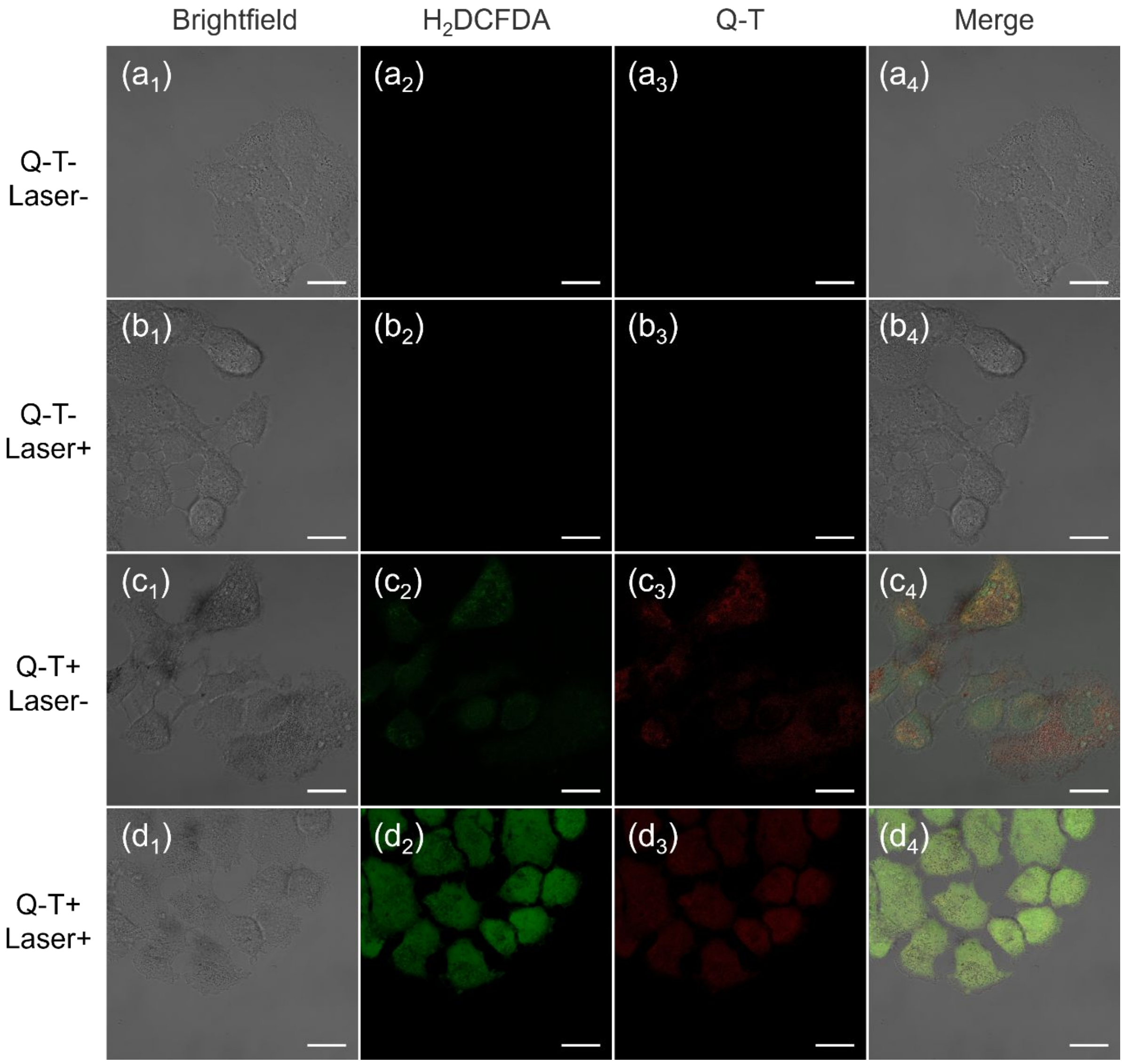

Figure S2. ROS assay *in vitro* with H_2DCFDA. Treatment for cells in each group, cells are cultured in normoxic conditions (21% oxygen level) with or without **Q-T** (2 μM) for 2 hours, and H_2DCFDA (5 μM) for 30 mins, followed with or without 532 nm laser irradiation (40 mW·cm^{-2}) for 5 mins. (a_1 - a_4). Q-T-/Laser-, (b_1 - b_4). Q-T-/Laser+,(c_1 - c_4). Q-T+/Laser-,(d_1 - d_4). Q-T+/Laser+. The scale bar is 20 μm.

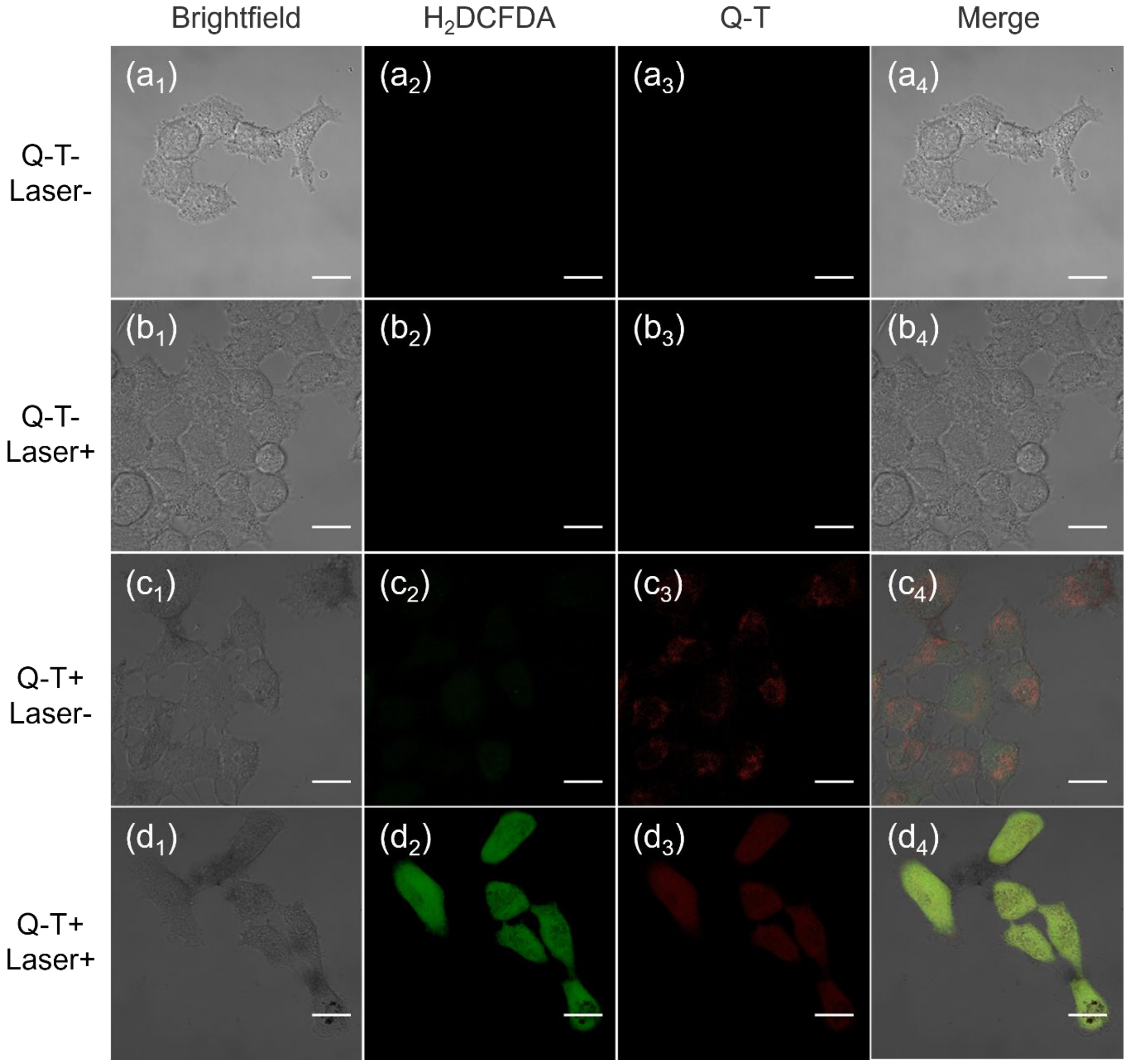

Figure S3. ROS assay *in vitro* with H_2DCFDA. Treatment for cells in each group, cells are cultured in hypoxic conditions (1% oxygen level) with or without **Q-T** (2 μM) for 2 hours, and H_2DCFDA (5 μM) for 30 mins, followed with or without 532 nm laser irradiation (40 mW·cm^{-2}) for 5 mins. (a_1 - a_4). Q-T-/Laser-, (b_1 - b_4). Q-T-/Laser+,(c_1 - c_4). Q-T+/Laser-,(d_1 - d_4). Q-T+/Laser+. The scale bar is 20 μm.

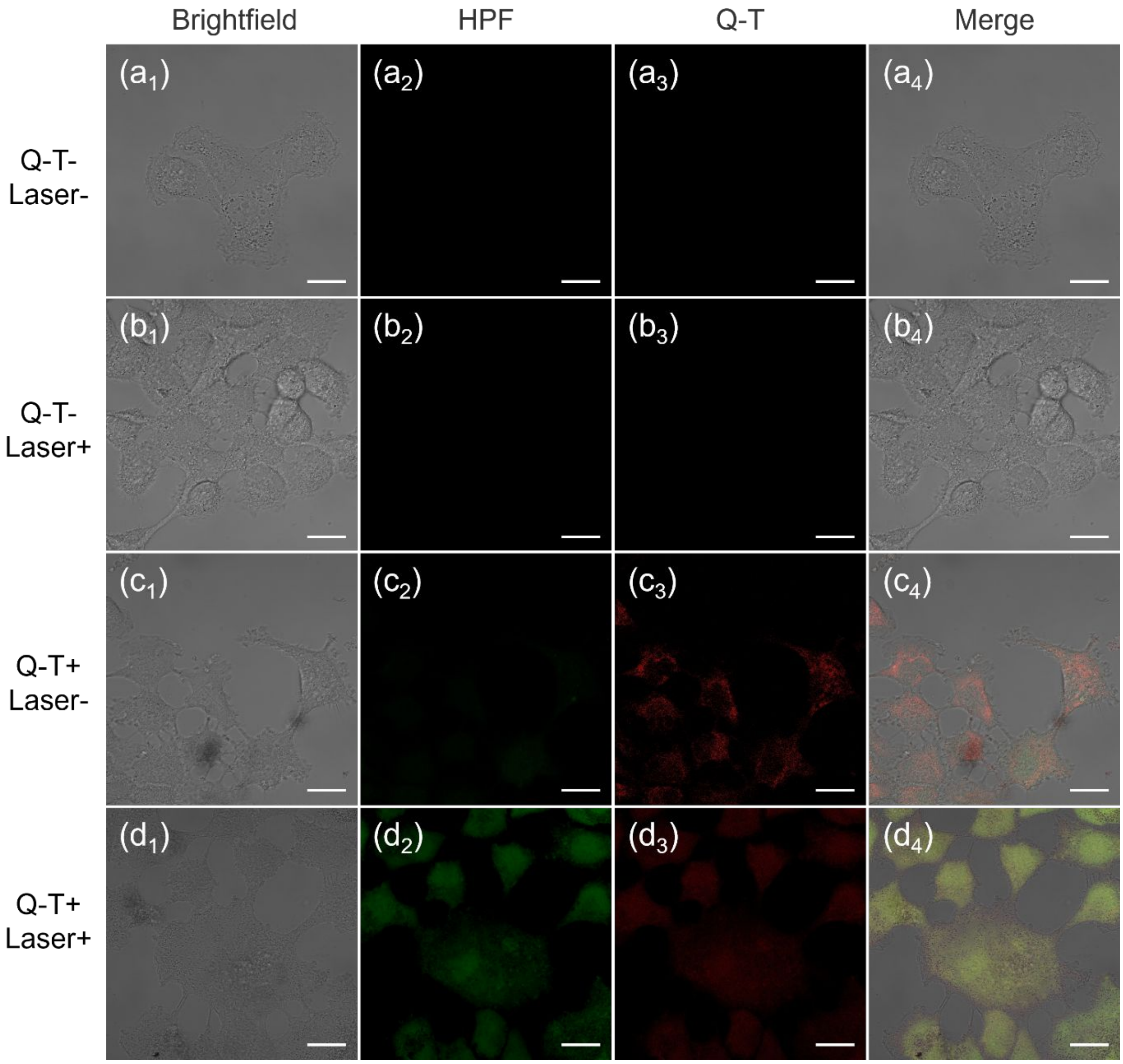

Figure S4. ROS assay *in vitro* with HPF. Treatment for cells in each group, cells are cultured with or without **Q-T** (2 μM) for 2 hours, and HPF (5 μM) for 30 mins, followed with or without 532 nm laser irradiation (40 mW·cm^{-2}) for 5 mins. (a_1 - a_4). Q-T-/Laser-, (b_1 - b_4). Q-T-/Laser+,(c_1 - c_4). Q-T+/Laser-,(d_1 - d_4). Q-T+/Laser+. The scale bar is 20 μm.

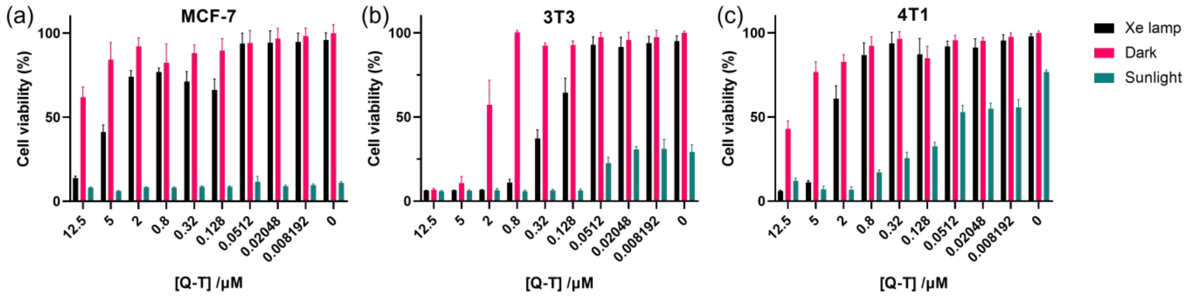

Figure S5. Photodynamic therapy for different cell lines with artificial and natural sunlight. (a). MCF-7 cells, (b). 3T3 cells, and (c). 4T1 cells. Experimental conditions: the irradiation groups were irradiated with Xe lamp or sunlight for 20 minutes.

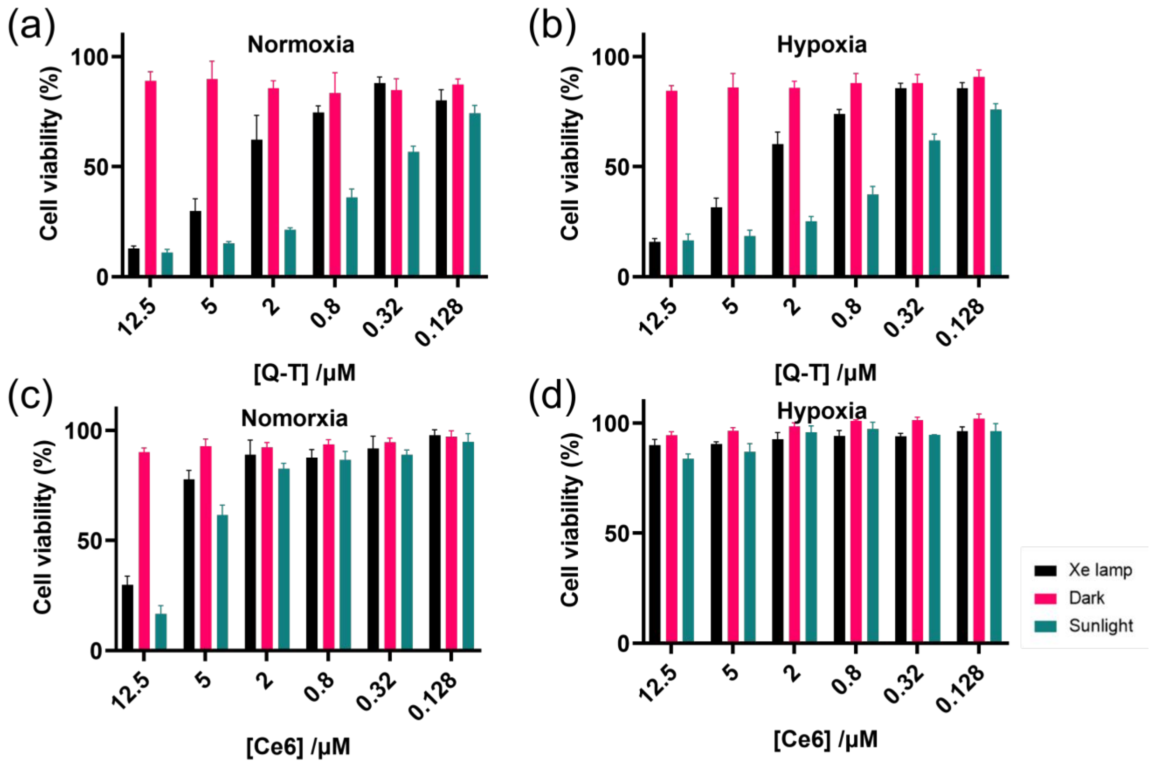

Figure S6. Photodynamic therapy for A431 cell lines in normoxic (21% oxygen level) and hypoxic conditions (1% oxygen level). (a). Q-T in normoxic condition, (b). Q-T in hypoxic condition, (c). Ce6 in normoxic condition, (d) Ce6 in hypoxic condition. Experimental conditions: the irradiation groups were irradiated with Xe lamp or sunlight for 20 minutes.

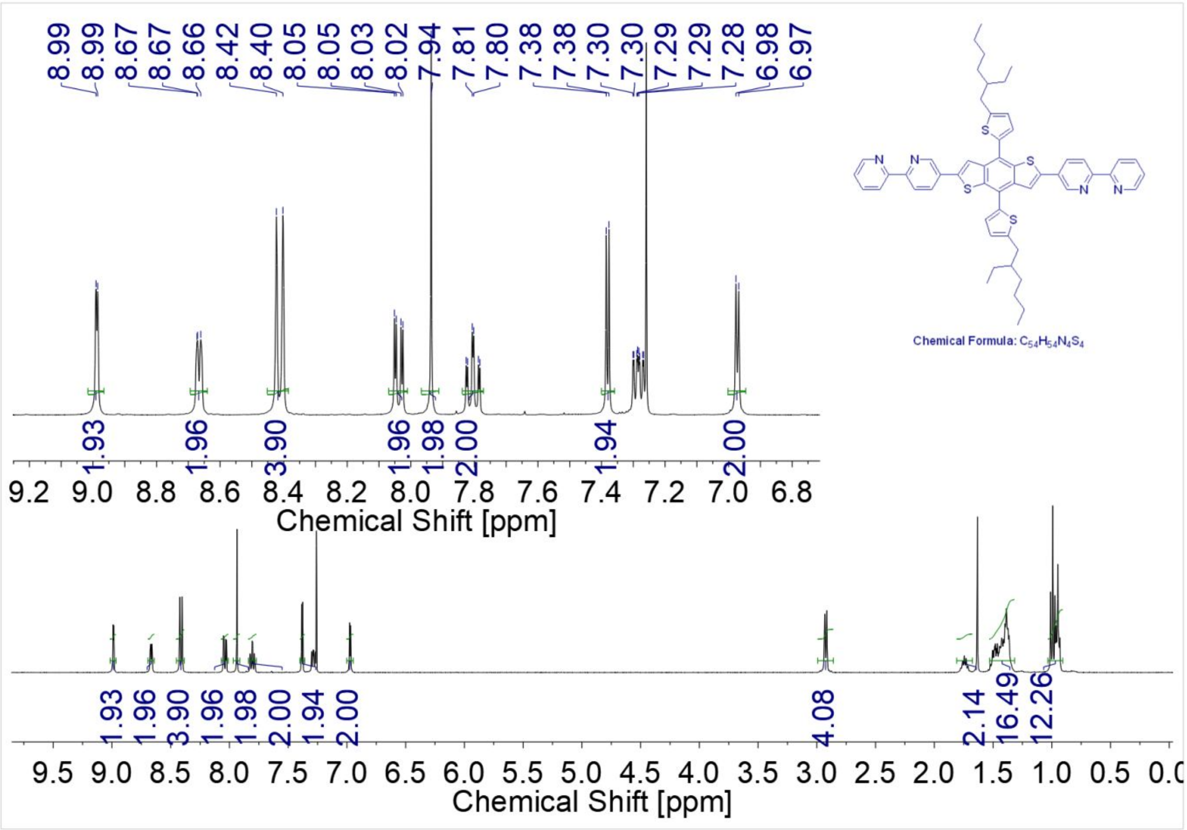

Figure S7. ^{1}HNMR spectrum of Pyd-T.

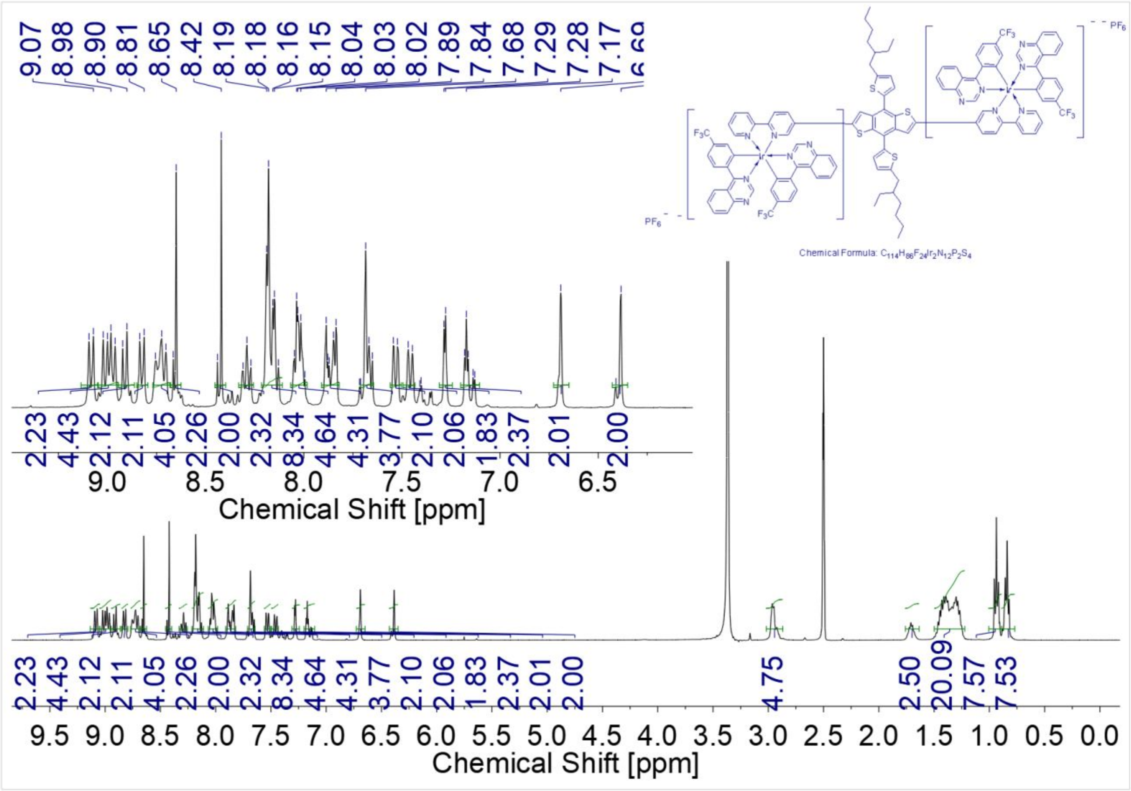

Figure S8. ^{1}HNMR spectrum of Q-T.

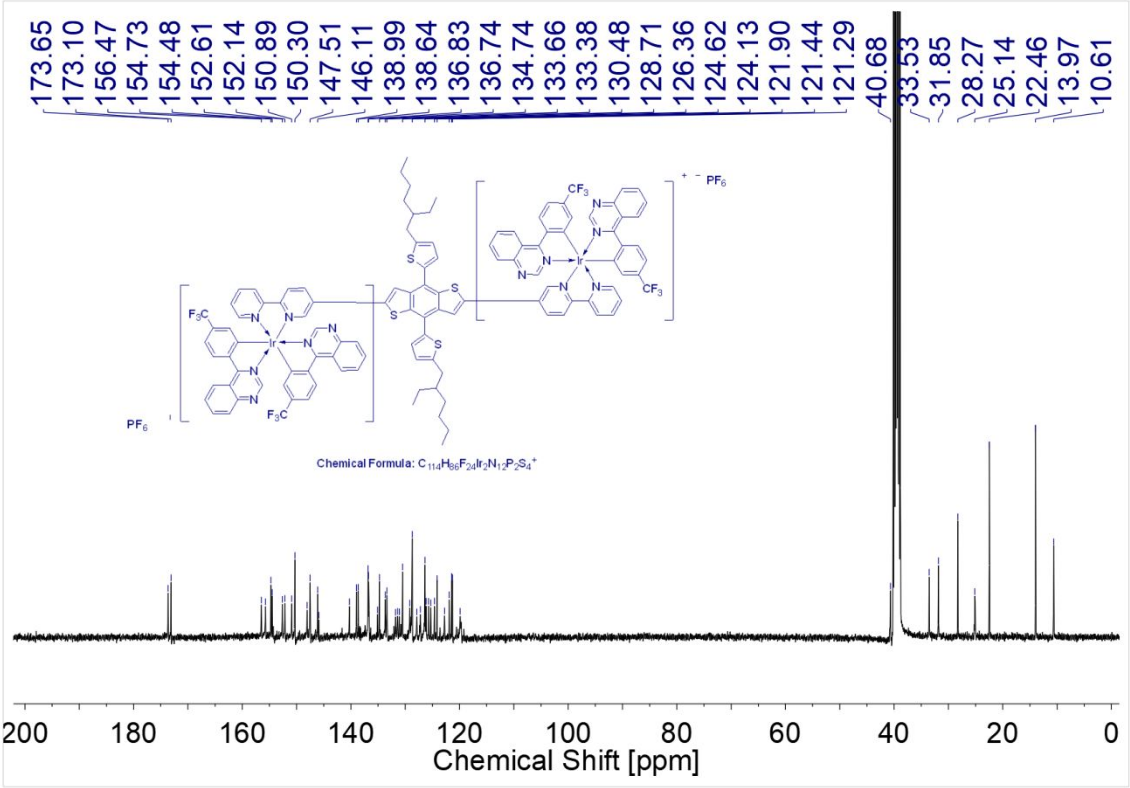

Figure S9. ^{13}CNMR spectrum of Q-T.

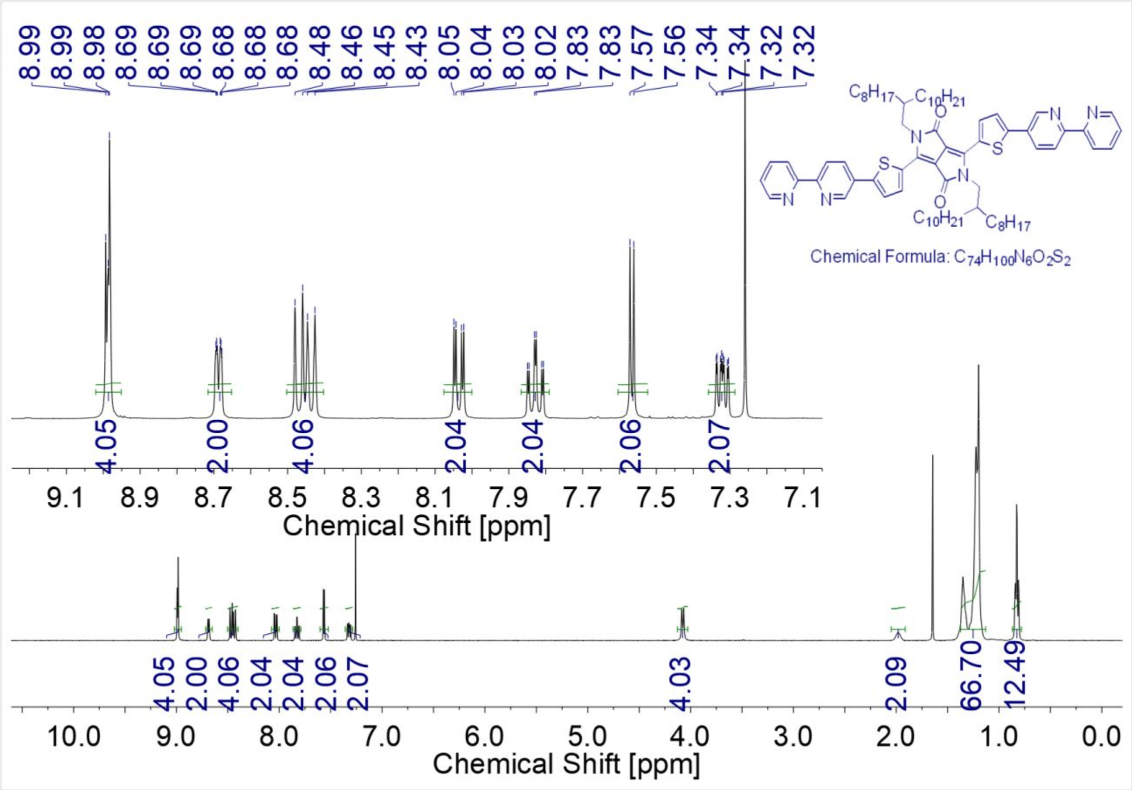

Figure S10. ^{1}HNMR spectrum of Pyd-D.

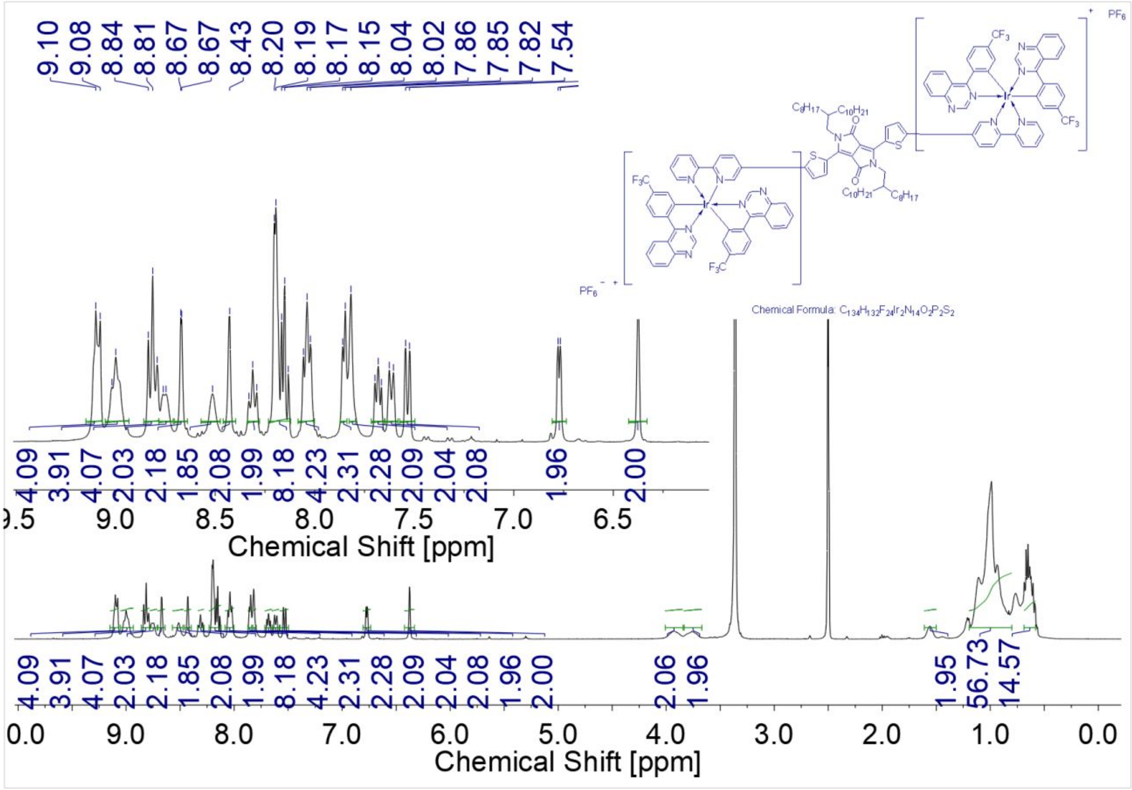

Figure S11. ^{1}HNMR spectrum of Q-D.

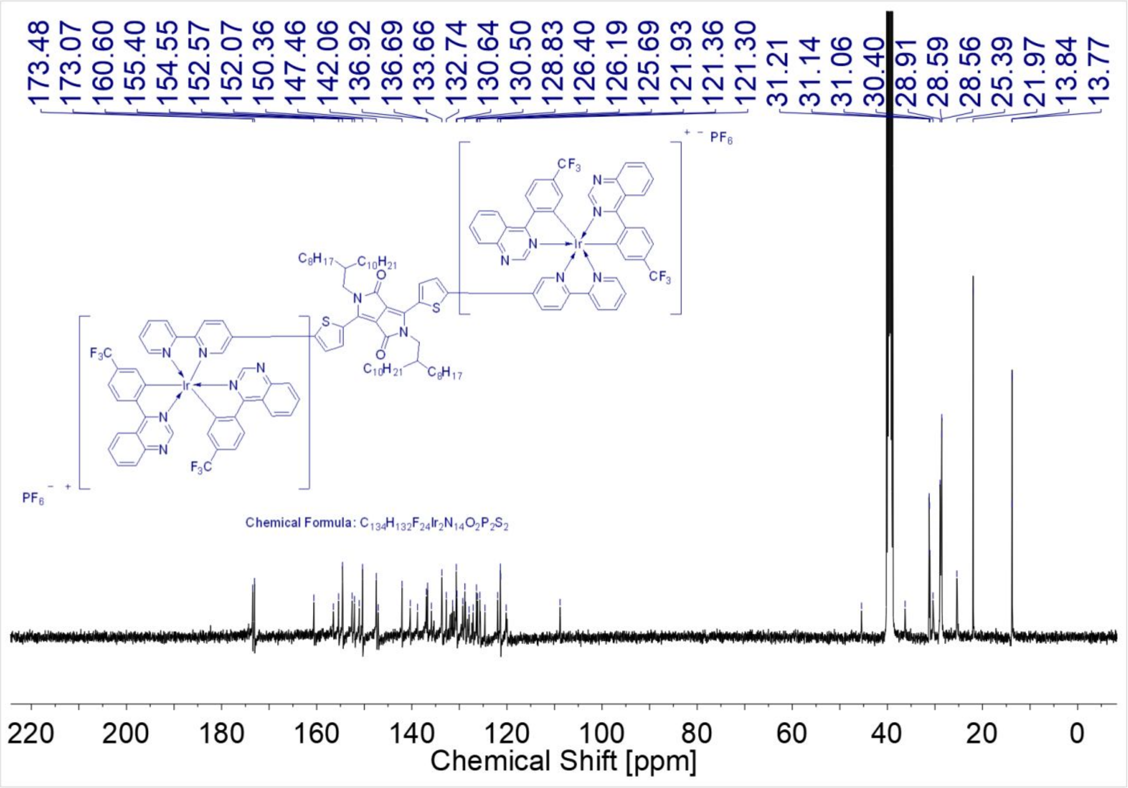

Figure S12. ^{13}CNMR spectrum of Q-D.

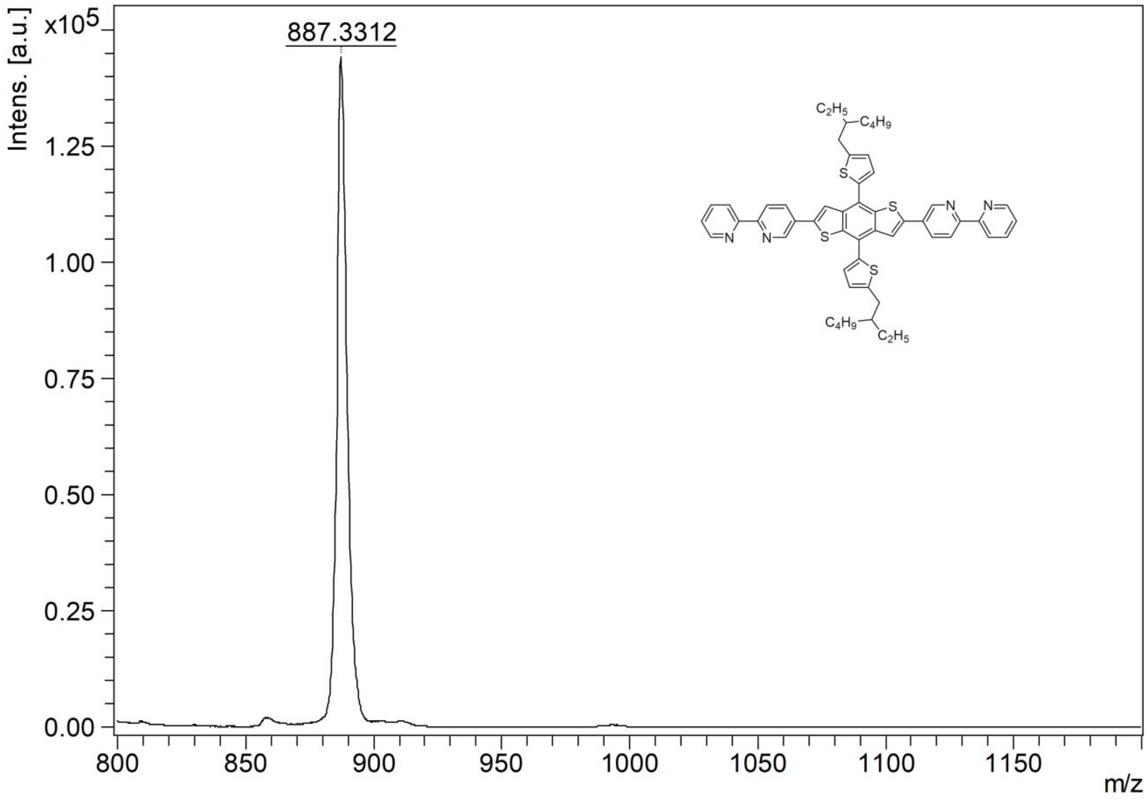

Figure S13. Maldi-ToF spectrum of Pyd-T.

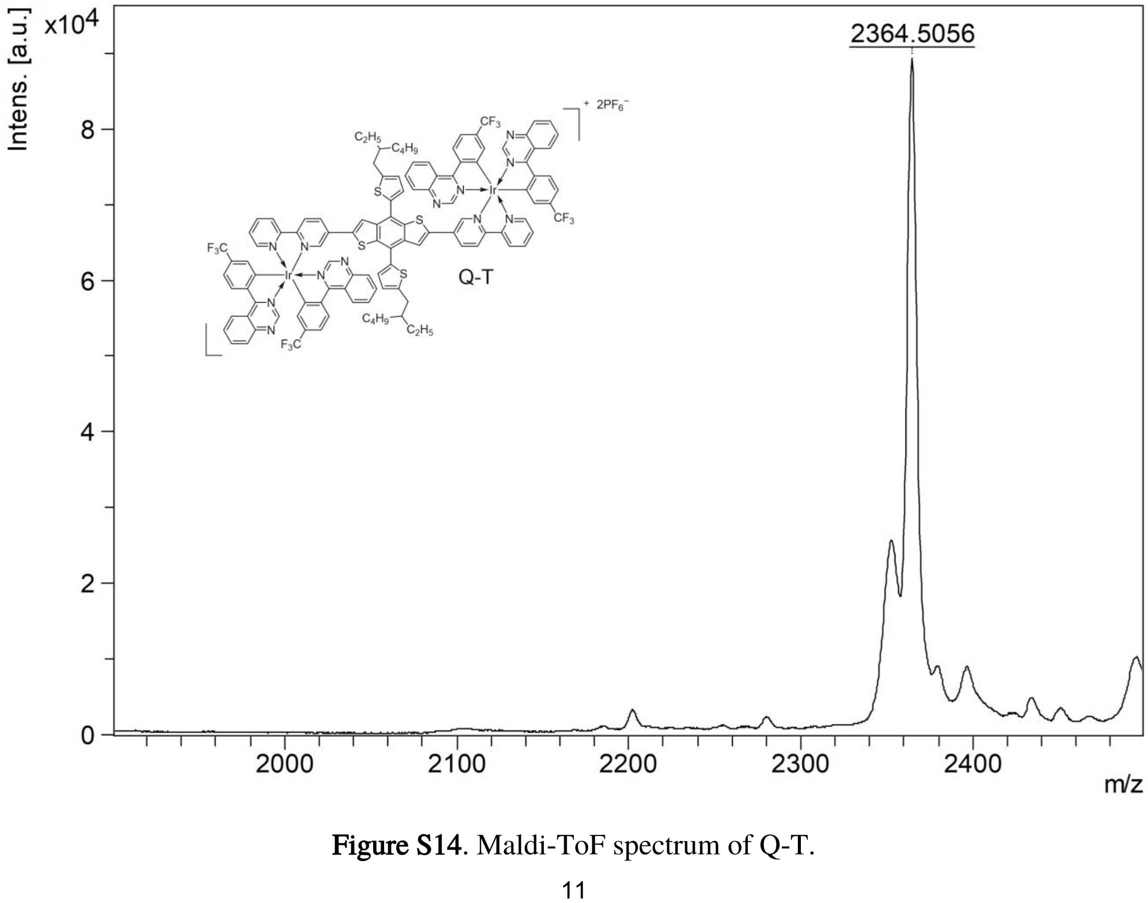

Figure S14. Maldi-ToF spectrum of Q-T.

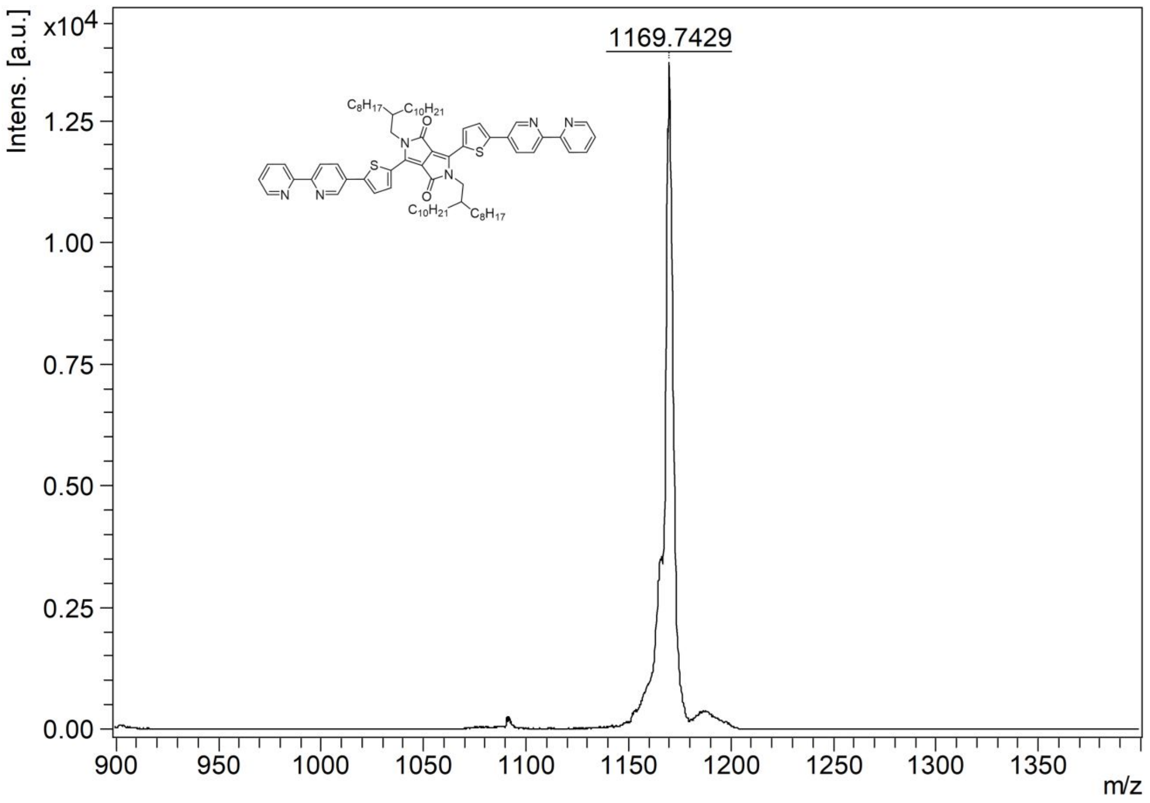

Figure S15. Maldi-ToF spectrum of Pyd-D.

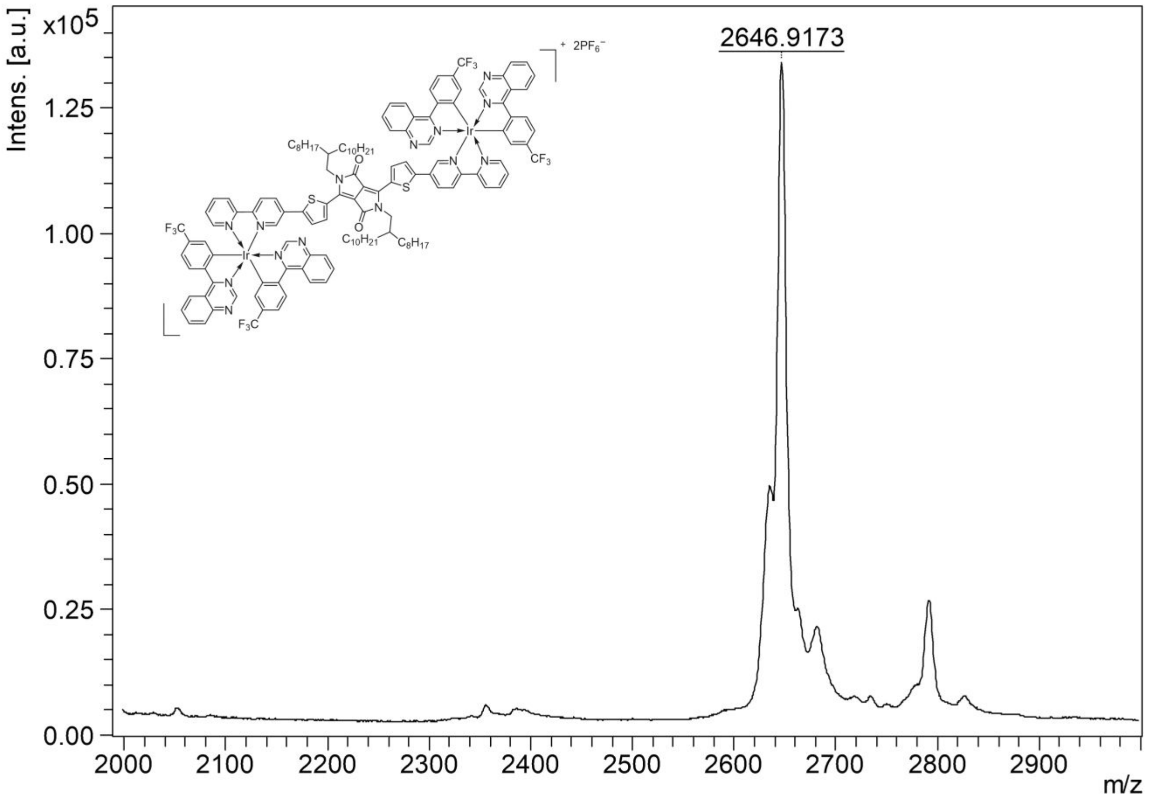

Figure S16. Maldi-ToF spectrum of Q-D.

Table S1. Mean Value and Error Limits for Data used in Figure 3(a)

Concentration [μM]	Xe Lamp		Dark		Sunlight	
	Mean Value	Erro Limits	Mean Value	Erro Limits	Mean Value	Erro Limits
12.5	12.78569	±1.1668	88.62643	±4.85486	11.54081	±1.29022
5	29.81891	±5.6531	89.21068	±7.86494	15.25094	±0.75635
2	62.81349	±9.7665	85.67287	±3.02741	21.43117	±0.66987
0.8	81.75358	±12.8043	83.3902	±8.36126	35.99411	±3.80971
0.32	87.47099	±2.4057	84.82057	±5.00248	56.75432	±2.59141
0.128	80.17983	±4.5778	87.18734	±2.32857	74.67717	±3.52706
0.051	80.07183	±3.8946	88.68434	±3.39427	84.8415	±1.56235
0.02	81.99158	±4.7952	89.82734	±4.51682	88.18177	±5.48408
0.008	82.59658	±4.5269	90.66984	±4.46278	87.60044	±5.12718
0	97.64443	±6.8021	100.2775	±4.11091	94.06295	±2.77613

List S1. Composition of each atom in HOMO of Q-T:

Atom	1(C) :	2.57206 %
Atom	2(C) :	2.91595 %
Atom	3(C) :	6.10322 %
Atom	4(C) :	6.27209 %
Atom	5(H) :	0.01717 %
Atom	6(C) :	9.40529 %
Atom	7(C) :	9.46230 %
Atom	8(C) :	6.20169 %
Atom	9(C) :	6.32682 %
Atom	10(S) :	5.72937 %
Atom	11(C) :	4.58945 %
Atom	12(C) :	1.82676 %
Atom	13(S) :	0.70112 %
Atom	14(C) :	1.02753 %
Atom	15(H) :	0.00436 %
Atom	16(C) :	2.74991 %
Atom	17(H) :	0.00571 %
Atom	18(C) :	4.82141 %
Atom	19(C) :	1.82442 %
Atom	20(S) :	0.70180 %
Atom	21(C) :	1.00764 %
Atom	22(H) :	0.00442 %
Atom	23(C) :	2.71321 %
Atom	24(H) :	0.00570 %
Atom	25(C) :	2.23045 %
Atom	26(C) :	3.04403 %
Atom	27(H) :	0.01807 %
Atom	28(S) :	5.71908 %
Atom	29(C) :	0.16261 %
Atom	30(H) :	0.00976 %
Atom	31(H) :	0.26179 %
Atom	32(H) :	0.30749 %
Atom	33(C) :	0.16253 %
Atom	34(H) :	0.26265 %
Atom	35(H) :	0.30013 %
Atom	36(H) :	0.00899 %
Atom	37(C) :	0.71380 %
Atom	38(C) :	0.66031 %

Atom	39(C) :	0.31203 %
Atom	40(N) :	0.08522 %
Atom	41(H) :	0.00113 %
Atom	42(C) :	0.13472 %
Atom	43(H) :	0.00054 %
Atom	44(Ir) :	0.37132 %
Atom	45(C) :	0.41706 %
Atom	46(H) :	0.00061 %
Atom	47(N) :	0.00314 %
Atom	48(C) :	0.10789 %
Atom	49(N) :	0.09450 %
Atom	50(C) :	0.14904 %
Atom	51(N) :	0.02505 %
Atom	52(C) :	0.00915 %
Atom	53(C) :	0.36651 %
Atom	54(C) :	0.01039 %
Atom	55(C) :	0.07927 %
Atom	56(C) :	0.13454 %
Atom	57(C) :	0.02664 %
Atom	58(C) :	0.24508 %
Atom	59(C) :	0.02144 %
Atom	60(C) :	0.02690 %
Atom	61(C) :	0.02287 %
Atom	62(C) :	0.14111 %
Atom	63(C) :	0.19945 %
Atom	64(H) :	0.00090 %
Atom	65(N) :	0.00305 %
Atom	66(C) :	0.01664 %
Atom	67(H) :	0.00001 %
Atom	68(C) :	0.00524 %
Atom	69(H) :	0.00015 %
Atom	70(C) :	0.12754 %
Atom	71(C) :	0.03694 %
Atom	72(C) :	0.03311 %
Atom	73(H) :	0.00091 %
Atom	74(C) :	0.02561 %
Atom	75(H) :	0.00037 %
Atom	76(N) :	0.00459 %
Atom	77(C) :	0.04628 %

Atom	78(H) :	0.00030 %
Atom	79(C) :	0.01651 %
Atom	80(C) :	0.01365 %
Atom	81(C) :	0.00490 %
Atom	82(H) :	0.00009 %
Atom	83(C) :	0.00082 %
Atom	84(H) :	0.00015 %
Atom	85(H) :	0.00831 %
Atom	86(C) :	0.13214 %
Atom	87(C) :	0.01573 %
Atom	88(C) :	0.02096 %
Atom	89(C) :	0.00567 %
Atom	90(H) :	0.00001 %
Atom	91(C) :	0.00795 %
Atom	92(C) :	0.00471 %
Atom	93(H) :	0.00011 %
Atom	94(H) :	0.00000 %
Atom	95(F) :	0.00003 %
Atom	96(F) :	0.00005 %
Atom	97(F) :	0.00002 %
Atom	98(H) :	0.00152 %
Atom	99(F) :	0.00027 %
Atom	100(F) :	0.00382 %
Atom	101(F) :	0.00108 %
Atom	102(C) :	0.00714 %
Atom	103(H) :	0.00003 %
Atom	104(C) :	0.00011 %
Atom	105(H) :	0.00002 %
Atom	106(C) :	0.00212 %
Atom	107(H) :	0.00001 %
Atom	108(C) :	0.00038 %
Atom	109(H) :	0.00003 %
Atom	110(H) :	0.00000 %
Atom	111(H) :	0.00000 %
Atom	112(H) :	0.00000 %
Atom	113(C) :	1.16357 %
Atom	114(C) :	0.72126 %
Atom	115(C) :	0.27733 %
Atom	116(N) :	0.10885 %

Atom	117(H) :	0.00134 %
Atom	118(C) :	0.08000 %
Atom	119(H) :	0.00042 %
Atom	120(Ir) :	0.47550 %
Atom	121(C) :	0.42090 %
Atom	122(H) :	0.00052 %
Atom	123(N) :	0.00375 %
Atom	124(C) :	0.10662 %
Atom	125(N) :	0.09599 %
Atom	126(C) :	0.20297 %
Atom	127(N) :	0.02517 %
Atom	128(C) :	0.01898 %
Atom	129(C) :	0.35553 %
Atom	130(C) :	0.00960 %
Atom	131(C) :	0.07800 %
Atom	132(C) :	0.13573 %
Atom	133(C) :	0.02653 %
Atom	134(C) :	0.30312 %
Atom	135(C) :	0.05841 %
Atom	136(C) :	0.02624 %
Atom	137(C) :	0.02201 %
Atom	138(C) :	0.13207 %
Atom	139(C) :	0.15456 %
Atom	140(H) :	0.00074 %
Atom	141(N) :	0.00288 %
Atom	142(C) :	0.01912 %
Atom	143(H) :	0.00001 %
Atom	144(C) :	0.00621 %
Atom	145(H) :	0.00018 %
Atom	146(C) :	0.12758 %
Atom	147(C) :	0.05614 %
Atom	148(C) :	0.05892 %
Atom	149(H) :	0.00085 %
Atom	150(C) :	0.02369 %
Atom	151(H) :	0.00038 %
Atom	152(N) :	0.00364 %
Atom	153(C) :	0.03561 %
Atom	154(H) :	0.00027 %
Atom	155(C) :	0.01376 %

Atom 156(C) : 0.00713 %
Atom 157(C) : 0.00540 %
Atom 158(H) : 0.00009 %
Atom 159(C) : 0.00130 %
Atom 160(H) : 0.00014 %
Atom 161(H) : 0.00944 %
Atom 162(C) : 0.16812 %
Atom 163(C) : 0.01825 %
Atom 164(C) : 0.01955 %
Atom 165(C) : 0.00581 %
Atom 166(H) : 0.00001 %
Atom 167(C) : 0.00525 %
Atom 168(C) : 0.00303 %
Atom 169(H) : 0.00008 %
Atom 170(H) : 0.00000 %
Atom 171(F) : 0.00002 %
Atom 172(F) : 0.00008 %
Atom 173(F) : 0.00002 %
Atom 174(H) : 0.00101 %
Atom 175(F) : 0.00029 %
Atom 176(F) : 0.00353 %
Atom 177(F) : 0.00099 %
Atom 178(C) : 0.00666 %
Atom 179(H) : 0.00003 %
Atom 180(C) : 0.00011 %
Atom 181(H) : 0.00002 %
Atom 182(C) : 0.00156 %
Atom 183(H) : 0.00001 %
Atom 184(C) : 0.00041 %
Atom 185(H) : 0.00002 %
Atom 186(H) : 0.00000 %
Atom 187(H) : 0.00000 %
Atom 188(H) : 0.00000 %

List S2. Composition of each atom in LUMO of Q-T:

Atom 1(C) : 1.17342 %
Atom 2(C) : 0.86785 %
Atom 3(C) : 0.61080 %
Atom 4(C) : 0.77577 %

Atom	5(H) :	0.00100 %
Atom	6(C) :	2.20615 %
Atom	7(C) :	1.97618 %
Atom	8(C) :	0.83848 %
Atom	9(C) :	0.50412 %
Atom	10(S) :	0.65363 %
Atom	11(C) :	0.85611 %
Atom	12(C) :	1.09048 %
Atom	13(S) :	0.13403 %
Atom	14(C) :	0.41190 %
Atom	15(H) :	0.00031 %
Atom	16(C) :	0.34462 %
Atom	17(H) :	0.00127 %
Atom	18(C) :	0.95241 %
Atom	19(C) :	1.07867 %
Atom	20(S) :	0.13227 %
Atom	21(C) :	0.41704 %
Atom	22(H) :	0.00035 %
Atom	23(C) :	0.34624 %
Atom	24(H) :	0.00145 %
Atom	25(C) :	1.19629 %
Atom	26(C) :	0.88607 %
Atom	27(H) :	0.00120 %
Atom	28(S) :	0.65130 %
Atom	29(C) :	0.00792 %
Atom	30(H) :	0.00035 %
Atom	31(H) :	0.00586 %
Atom	32(H) :	0.00754 %
Atom	33(C) :	0.00776 %
Atom	34(H) :	0.00576 %
Atom	35(H) :	0.00724 %
Atom	36(H) :	0.00035 %
Atom	37(C) :	2.85344 %
Atom	38(C) :	1.12227 %
Atom	39(C) :	1.19282 %
Atom	40(N) :	0.79177 %
Atom	41(H) :	0.00092 %
Atom	42(C) :	0.55651 %
Atom	43(H) :	0.00119 %

Atom	44(Ir) :	3.93787 %
Atom	45(C) :	2.09440 %
Atom	46(H) :	0.00055 %
Atom	47(N) :	1.04500 %
Atom	48(C) :	0.84589 %
Atom	49(N) :	1.06657 %
Atom	50(C) :	2.24051 %
Atom	51(N) :	0.80566 %
Atom	52(C) :	0.91308 %
Atom	53(C) :	2.56312 %
Atom	54(C) :	0.31902 %
Atom	55(C) :	0.70098 %
Atom	56(C) :	1.04077 %
Atom	57(C) :	0.28471 %
Atom	58(C) :	1.06278 %
Atom	59(C) :	0.54894 %
Atom	60(C) :	2.32370 %
Atom	61(C) :	0.31159 %
Atom	62(C) :	0.71555 %
Atom	63(C) :	0.50539 %
Atom	64(H) :	0.00351 %
Atom	65(N) :	0.72747 %
Atom	66(C) :	0.38015 %
Atom	67(H) :	0.00040 %
Atom	68(C) :	0.49036 %
Atom	69(H) :	0.00144 %
Atom	70(C) :	1.24945 %
Atom	71(C) :	0.57258 %
Atom	72(C) :	0.69616 %
Atom	73(H) :	0.00018 %
Atom	74(C) :	0.62305 %
Atom	75(H) :	0.00362 %
Atom	76(N) :	0.59734 %
Atom	77(C) :	0.54298 %
Atom	78(H) :	0.00063 %
Atom	79(C) :	0.32981 %
Atom	80(C) :	0.89465 %
Atom	81(C) :	0.07013 %
Atom	82(H) :	0.00351 %

Atom	83(C) :	0.04062 %
Atom	84(H) :	0.00116 %
Atom	85(H) :	0.01104 %
Atom	86(C) :	0.18006 %
Atom	87(C) :	0.06512 %
Atom	88(C) :	0.39704 %
Atom	89(C) :	1.09126 %
Atom	90(H) :	0.00046 %
Atom	91(C) :	0.31172 %
Atom	92(C) :	0.34973 %
Atom	93(H) :	0.00547 %
Atom	94(H) :	0.00094 %
Atom	95(F) :	0.01217 %
Atom	96(F) :	0.05729 %
Atom	97(F) :	0.02551 %
Atom	98(H) :	0.00269 %
Atom	99(F) :	0.02335 %
Atom	100(F) :	0.02549 %
Atom	101(F) :	0.07754 %
Atom	102(C) :	0.27334 %
Atom	103(H) :	0.00551 %
Atom	104(C) :	0.37428 %
Atom	105(H) :	0.00052 %
Atom	106(C) :	0.32412 %
Atom	107(H) :	0.00101 %
Atom	108(C) :	0.27355 %
Atom	109(H) :	0.00051 %
Atom	110(H) :	0.00082 %
Atom	111(H) :	0.00046 %
Atom	112(H) :	0.00039 %
Atom	113(C) :	2.81277 %
Atom	114(C) :	1.06700 %
Atom	115(C) :	1.17962 %
Atom	116(N) :	0.78556 %
Atom	117(H) :	0.00108 %
Atom	118(C) :	0.51976 %
Atom	119(H) :	0.00121 %
Atom	120(Ir) :	3.94666 %
Atom	121(C) :	2.10504 %

Atom	122(H) :	0.00054 %
Atom	123(N) :	1.02053 %
Atom	124(C) :	0.86036 %
Atom	125(N) :	1.05581 %
Atom	126(C) :	2.62095 %
Atom	127(N) :	0.76043 %
Atom	128(C) :	0.92757 %
Atom	129(C) :	2.40628 %
Atom	130(C) :	0.31672 %
Atom	131(C) :	0.63099 %
Atom	132(C) :	1.00230 %
Atom	133(C) :	0.27924 %
Atom	134(C) :	1.24990 %
Atom	135(C) :	0.76361 %
Atom	136(C) :	2.19834 %
Atom	137(C) :	0.31966 %
Atom	138(C) :	0.71998 %
Atom	139(C) :	0.50854 %
Atom	140(H) :	0.00301 %
Atom	141(N) :	0.70717 %
Atom	142(C) :	0.36166 %
Atom	143(H) :	0.00048 %
Atom	144(C) :	0.41082 %
Atom	145(H) :	0.00177 %
Atom	146(C) :	1.25853 %
Atom	147(C) :	0.56442 %
Atom	148(C) :	0.75342 %
Atom	149(H) :	0.00017 %
Atom	150(C) :	0.58507 %
Atom	151(H) :	0.00355 %
Atom	152(N) :	0.55888 %
Atom	153(C) :	0.53321 %
Atom	154(H) :	0.00063 %
Atom	155(C) :	0.31405 %
Atom	156(C) :	0.85574 %
Atom	157(C) :	0.08172 %
Atom	158(H) :	0.00342 %
Atom	159(C) :	0.03968 %
Atom	160(H) :	0.00116 %

Atom	161(H) :	0.01130 %
Atom	162(C) :	0.18879 %
Atom	163(C) :	0.06432 %
Atom	164(C) :	0.37183 %
Atom	165(C) :	1.05084 %
Atom	166(H) :	0.00047 %
Atom	167(C) :	0.31050 %
Atom	168(C) :	0.33800 %
Atom	169(H) :	0.00521 %
Atom	170(H) :	0.00098 %
Atom	171(F) :	0.00818 %
Atom	172(F) :	0.04901 %
Atom	173(F) :	0.03250 %
Atom	174(H) :	0.00226 %
Atom	175(F) :	0.02006 %
Atom	176(F) :	0.02712 %
Atom	177(F) :	0.07523 %
Atom	178(C) :	0.26933 %
Atom	179(H) :	0.00536 %
Atom	180(C) :	0.37122 %
Atom	181(H) :	0.00056 %
Atom	182(C) :	0.31539 %
Atom	183(H) :	0.00095 %
Atom	184(C) :	0.26174 %
Atom	185(H) :	0.00049 %
Atom	186(H) :	0.00075 %
Atom	187(H) :	0.00044 %
Atom	188(H) :	0.00038 %

List 3. Composition of each atom in HOMO of Q-D:

Atom	1(Ir) :	0.24842 %
Atom	2(C) :	0.33877 %
Atom	3(C) :	0.04819 %
Atom	4(C) :	0.31578 %
Atom	5(C) :	1.38050 %
Atom	6(C) :	0.30911 %
Atom	7(H) :	0.00004 %
Atom	8(C) :	0.05499 %
Atom	9(H) :	0.00035 %

Atom	10(C) :	1.42795 %
Atom	11(H) :	0.00035 %
Atom	12(H) :	0.00002 %
Atom	13(C) :	1.10060 %
Atom	14(C) :	2.16912 %
Atom	15(H) :	0.00122 %
Atom	16(C) :	0.95026 %
Atom	17(H) :	0.00401 %
Atom	18(H) :	0.00187 %
Atom	19(N) :	0.31332 %
Atom	20(N) :	0.05040 %
Atom	21(C) :	0.03084 %
Atom	22(C) :	0.00942 %
Atom	23(C) :	0.00239 %
Atom	24(C) :	0.04533 %
Atom	25(H) :	0.00001 %
Atom	26(C) :	0.00221 %
Atom	27(C) :	0.25494 %
Atom	28(C) :	0.03055 %
Atom	29(C) :	0.60986 %
Atom	30(C) :	0.01578 %
Atom	31(H) :	0.00016 %
Atom	32(C) :	0.31312 %
Atom	33(H) :	0.00045 %
Atom	34(H) :	0.00001 %
Atom	35(C) :	0.27981 %
Atom	36(C) :	0.01344 %
Atom	37(C) :	0.11328 %
Atom	38(C) :	0.01657 %
Atom	39(H) :	0.00032 %
Atom	40(C) :	0.02254 %
Atom	41(C) :	0.01758 %
Atom	42(C) :	0.02830 %
Atom	43(C) :	0.07523 %
Atom	44(C) :	0.00656 %
Atom	45(H) :	0.00000 %
Atom	46(C) :	0.06583 %
Atom	47(H) :	0.00031 %
Atom	48(H) :	0.00001 %

Atom	49(N) :	0.00416 %
Atom	50(N) :	0.01463 %
Atom	51(N) :	0.00119 %
Atom	52(N) :	0.00483 %
Atom	53(C) :	0.00235 %
Atom	54(C) :	0.00211 %
Atom	55(F) :	0.00017 %
Atom	56(F) :	0.00023 %
Atom	57(F) :	0.00010 %
Atom	58(F) :	0.00041 %
Atom	59(F) :	0.00017 %
Atom	60(F) :	0.00028 %
Atom	61(C) :	0.00017 %
Atom	62(C) :	0.00023 %
Atom	63(H) :	0.00005 %
Atom	64(H) :	0.00000 %
Atom	65(C) :	0.00151 %
Atom	66(C) :	0.00013 %
Atom	67(H) :	0.00000 %
Atom	68(H) :	0.00000 %
Atom	69(C) :	0.01939 %
Atom	70(C) :	0.06135 %
Atom	71(H) :	0.00019 %
Atom	72(H) :	0.00138 %
Atom	73(C) :	0.08278 %
Atom	74(C) :	0.02241 %
Atom	75(H) :	0.00011 %
Atom	76(H) :	0.00002 %
Atom	77(C) :	4.53712 %
Atom	78(C) :	2.35860 %
Atom	79(C) :	1.33716 %
Atom	80(C) :	4.56629 %
Atom	81(H) :	0.00547 %
Atom	82(C) :	4.77433 %
Atom	83(C) :	1.39112 %
Atom	84(S) :	0.77680 %
Atom	85(Ir) :	0.24078 %
Atom	86(C) :	0.35205 %
Atom	87(C) :	0.04831 %

Atom	88(C) :	0.31284 %
Atom	89(C) :	1.39024 %
Atom	90(C) :	0.30416 %
Atom	91(H) :	0.00004 %
Atom	92(C) :	0.05763 %
Atom	93(H) :	0.00033 %
Atom	94(C) :	1.81356 %
Atom	95(H) :	0.00035 %
Atom	96(H) :	0.00001 %
Atom	97(C) :	1.19723 %
Atom	98(C) :	2.65328 %
Atom	99(H) :	0.00135 %
Atom	100(C) :	1.15055 %
Atom	101(H) :	0.00406 %
Atom	102(H) :	0.00224 %
Atom	103(N) :	0.30839 %
Atom	104(N) :	0.05745 %
Atom	105(C) :	0.02704 %
Atom	106(C) :	0.00987 %
Atom	107(C) :	0.00367 %
Atom	108(C) :	0.04938 %
Atom	109(H) :	0.00000 %
Atom	110(C) :	0.00244 %
Atom	111(C) :	0.30661 %
Atom	112(C) :	0.03339 %
Atom	113(C) :	0.67323 %
Atom	114(C) :	0.01672 %
Atom	115(H) :	0.00014 %
Atom	116(C) :	0.33967 %
Atom	117(H) :	0.00055 %
Atom	118(H) :	0.00001 %
Atom	119(C) :	0.26680 %
Atom	120(C) :	0.01579 %
Atom	121(C) :	0.13309 %
Atom	122(C) :	0.02204 %
Atom	123(H) :	0.00036 %
Atom	124(C) :	0.03189 %
Atom	125(C) :	0.01596 %
Atom	126(C) :	0.02645 %

Atom	127(C) :	0.06535 %
Atom	128(C) :	0.00627 %
Atom	129(H) :	0.00000 %
Atom	130(C) :	0.06315 %
Atom	131(H) :	0.00029 %
Atom	132(H) :	0.00000 %
Atom	133(N) :	0.00378 %
Atom	134(N) :	0.01476 %
Atom	135(N) :	0.00120 %
Atom	136(N) :	0.00551 %
Atom	137(C) :	0.00310 %
Atom	138(C) :	0.00193 %
Atom	139(F) :	0.00013 %
Atom	140(F) :	0.00032 %
Atom	141(F) :	0.00008 %
Atom	142(F) :	0.00039 %
Atom	143(F) :	0.00015 %
Atom	144(F) :	0.00027 %
Atom	145(C) :	0.00026 %
Atom	146(C) :	0.00021 %
Atom	147(H) :	0.00007 %
Atom	148(H) :	0.00000 %
Atom	149(C) :	0.00134 %
Atom	150(C) :	0.00008 %
Atom	151(H) :	0.00000 %
Atom	152(H) :	0.00000 %
Atom	153(C) :	0.02928 %
Atom	154(C) :	0.04321 %
Atom	155(H) :	0.00015 %
Atom	156(H) :	0.00062 %
Atom	157(C) :	0.07777 %
Atom	158(C) :	0.01400 %
Atom	159(H) :	0.00007 %
Atom	160(H) :	0.00004 %
Atom	161(C) :	4.65718 %
Atom	162(C) :	2.72873 %
Atom	163(H) :	0.00566 %
Atom	164(S) :	0.77932 %
Atom	165(H) :	0.00285 %

Atom	166(H) :	0.00295 %
Atom	167(C) :	4.41323 %
Atom	168(C) :	9.98373 %
Atom	169(C) :	10.04136 %
Atom	170(C) :	4.42924 %
Atom	171(C) :	1.82756 %
Atom	172(O) :	6.29338 %
Atom	173(C) :	1.83517 %
Atom	174(O) :	6.28535 %
Atom	175(N) :	1.69453 %
Atom	176(N) :	1.69159 %
Atom	177(C) :	0.08165 %
Atom	178(H) :	0.08144 %
Atom	179(H) :	0.08032 %
Atom	180(H) :	0.00022 %
Atom	181(C) :	0.08326 %
Atom	182(H) :	0.00014 %
Atom	183(H) :	0.08119 %
Atom	184(H) :	0.07883 %

List 4. Composition of each atom in LUMO of Q-D:

Atom	1(Ir) :	1.95200 %
Atom	2(C) :	0.84281 %
Atom	3(C) :	0.08674 %
Atom	4(C) :	0.72077 %
Atom	5(C) :	2.66475 %
Atom	6(C) :	0.99908 %
Atom	7(H) :	0.00065 %
Atom	8(C) :	0.25137 %
Atom	9(H) :	0.00080 %
Atom	10(C) :	1.72552 %
Atom	11(H) :	0.00109 %
Atom	12(H) :	0.00025 %
Atom	13(C) :	0.72971 %
Atom	14(C) :	2.37811 %
Atom	15(H) :	0.00148 %
Atom	16(C) :	3.76750 %
Atom	17(H) :	0.00673 %
Atom	18(H) :	0.00407 %

Atom	19(N) :	1.14158 %
Atom	20(N) :	0.46123 %
Atom	21(C) :	0.15097 %
Atom	22(C) :	0.08348 %
Atom	23(C) :	0.07317 %
Atom	24(C) :	0.17974 %
Atom	25(H) :	0.00181 %
Atom	26(C) :	0.07252 %
Atom	27(C) :	0.32629 %
Atom	28(C) :	0.04113 %
Atom	29(C) :	0.22445 %
Atom	30(C) :	0.05638 %
Atom	31(H) :	0.00134 %
Atom	32(C) :	0.28152 %
Atom	33(H) :	0.00030 %
Atom	34(H) :	0.00004 %
Atom	35(C) :	1.24120 %
Atom	36(C) :	0.15438 %
Atom	37(C) :	0.16080 %
Atom	38(C) :	0.28078 %
Atom	39(H) :	0.00316 %
Atom	40(C) :	0.12309 %
Atom	41(C) :	0.17315 %
Atom	42(C) :	0.30580 %
Atom	43(C) :	0.18368 %
Atom	44(C) :	0.03205 %
Atom	45(H) :	0.00033 %
Atom	46(C) :	0.31929 %
Atom	47(H) :	0.00201 %
Atom	48(H) :	0.00012 %
Atom	49(N) :	0.08765 %
Atom	50(N) :	0.13262 %
Atom	51(N) :	0.02645 %
Atom	52(N) :	0.11438 %
Atom	53(C) :	0.01305 %
Atom	54(C) :	0.00853 %
Atom	55(F) :	0.00369 %
Atom	56(F) :	0.00110 %
Atom	57(F) :	0.00257 %

Atom	58(F) :	0.00557 %
Atom	59(F) :	0.00489 %
Atom	60(F) :	0.00130 %
Atom	61(C) :	0.02409 %
Atom	62(C) :	0.00942 %
Atom	63(H) :	0.00001 %
Atom	64(H) :	0.00008 %
Atom	65(C) :	0.04293 %
Atom	66(C) :	0.01202 %
Atom	67(H) :	0.00005 %
Atom	68(H) :	0.00003 %
Atom	69(C) :	0.10494 %
Atom	70(C) :	0.11944 %
Atom	71(H) :	0.00001 %
Atom	72(H) :	0.00080 %
Atom	73(C) :	0.11019 %
Atom	74(C) :	0.10182 %
Atom	75(H) :	0.00546 %
Atom	76(H) :	0.00006 %
Atom	77(C) :	4.95359 %
Atom	78(C) :	0.91728 %
Atom	79(C) :	1.28122 %
Atom	80(C) :	2.22053 %
Atom	81(H) :	0.00492 %
Atom	82(C) :	2.80239 %
Atom	83(C) :	1.37329 %
Atom	84(S) :	3.27947 %
Atom	85(Ir) :	1.90584 %
Atom	86(C) :	0.85375 %
Atom	87(C) :	0.08496 %
Atom	88(C) :	0.71563 %
Atom	89(C) :	2.65317 %
Atom	90(C) :	0.98212 %
Atom	91(H) :	0.00061 %
Atom	92(C) :	0.25468 %
Atom	93(H) :	0.00074 %
Atom	94(C) :	2.30675 %
Atom	95(H) :	0.00107 %
Atom	96(H) :	0.00022 %

Atom	97(C) :	0.74961 %
Atom	98(C) :	3.01001 %
Atom	99(H) :	0.00168 %
Atom	100(C) :	4.20593 %
Atom	101(H) :	0.00680 %
Atom	102(H) :	0.00539 %
Atom	103(N) :	1.12365 %
Atom	104(N) :	0.46354 %
Atom	105(C) :	0.14735 %
Atom	106(C) :	0.08472 %
Atom	107(C) :	0.07478 %
Atom	108(C) :	0.15296 %
Atom	109(H) :	0.00173 %
Atom	110(C) :	0.07069 %
Atom	111(C) :	0.37623 %
Atom	112(C) :	0.04454 %
Atom	113(C) :	0.26155 %
Atom	114(C) :	0.04268 %
Atom	115(H) :	0.00124 %
Atom	116(C) :	0.28422 %
Atom	117(H) :	0.00029 %
Atom	118(H) :	0.00004 %
Atom	119(C) :	1.15091 %
Atom	120(C) :	0.15745 %
Atom	121(C) :	0.15843 %
Atom	122(C) :	0.23794 %
Atom	123(H) :	0.00318 %
Atom	124(C) :	0.12413 %
Atom	125(C) :	0.17177 %
Atom	126(C) :	0.28409 %
Atom	127(C) :	0.15268 %
Atom	128(C) :	0.02999 %
Atom	129(H) :	0.00033 %
Atom	130(C) :	0.29216 %
Atom	131(H) :	0.00187 %
Atom	132(H) :	0.00012 %
Atom	133(N) :	0.08521 %
Atom	134(N) :	0.12790 %
Atom	135(N) :	0.02456 %

Atom	136(N) :	0.11602 %
Atom	137(C) :	0.01285 %
Atom	138(C) :	0.00807 %
Atom	139(F) :	0.00273 %
Atom	140(F) :	0.00165 %
Atom	141(F) :	0.00305 %
Atom	142(F) :	0.00564 %
Atom	143(F) :	0.00460 %
Atom	144(F) :	0.00134 %
Atom	145(C) :	0.02228 %
Atom	146(C) :	0.00805 %
Atom	147(H) :	0.00003 %
Atom	148(H) :	0.00008 %
Atom	149(C) :	0.03988 %
Atom	150(C) :	0.01081 %
Atom	151(H) :	0.00005 %
Atom	152(H) :	0.00003 %
Atom	153(C) :	0.11608 %
Atom	154(C) :	0.08837 %
Atom	155(H) :	0.00002 %
Atom	156(H) :	0.00036 %
Atom	157(C) :	0.09234 %
Atom	158(C) :	0.07220 %
Atom	159(H) :	0.00499 %
Atom	160(H) :	0.00003 %
Atom	161(C) :	5.14859 %
Atom	162(C) :	1.66895 %
Atom	163(H) :	0.00745 %
Atom	164(S) :	3.24894 %
Atom	165(H) :	0.00155 %
Atom	166(H) :	0.00158 %
Atom	167(C) :	6.94346 %
Atom	168(C) :	1.67829 %
Atom	169(C) :	1.16285 %
Atom	170(C) :	6.92461 %
Atom	171(C) :	1.17882 %
Atom	172(O) :	1.97652 %
Atom	173(C) :	1.18881 %
Atom	174(O) :	2.00113 %

Atom	175(N) :	1.09650 %
Atom	176(N) :	1.09156 %
Atom	177(C) :	0.03570 %
Atom	178(H) :	0.03229 %
Atom	179(H) :	0.04425 %
Atom	180(H) :	0.00032 %
Atom	181(C) :	0.03464 %
Atom	182(H) :	0.00035 %
Atom	183(H) :	0.03113 %
Atom	184(H) :	0.04322 %
